# Synergistic Modulation of Nitrogen and Chemical Regulation: Balancing Photosynthesis and Lodging Resistance for High-Efficiency Maize Production Under Dense Planting

**DOI:** 10.3390/plants15030500

**Published:** 2026-02-05

**Authors:** Xiaoming Liu, Yao Meng, Ling Dong, Yubo Hao, Yang Yu, Guoyi Lv, Yubo Jiang, Yiteng Zhang, Chunrong Qian, Wanrong Gu

**Affiliations:** 1 College of Biology and Agriculture, Jiamusi University, Jiamusi 154007, China; liuxiaoming0913@163.com; 2 College of Agronomy, Northeast Agricultural University, Harbin 150030, China; dongling@neau.edu.cn; 3 Scientific Research Management Department, Heilongjiang Academy of Land Reclamation Sciences, Harbin 150038, China; mengyao830922@163.com; 4 Institute of Crop Cultivation and Tillage, Heilongjiang Academy of Agricultural Sciences, Harbin 150086, China; yubohao@haas.cn (Y.H.); yuyanghaas@163.com (Y.Y.); guoyilv@haas.cn (G.L.); jiangyubo@haas.cn (Y.J.); zyt@haas.cn (Y.Z.)

**Keywords:** nitrogen fertilizer, chemical regulation, photosynthesis, lodging resistance, nitrogen use efficiency

## Abstract

While increasing planting density is a viable strategy for enhancing maize yield, it concurrently elevates the risks of lodging and accelerated leaf senescence due to intensified inter-plant competition, which can ultimately compromise yield stability. A field experiment was conducted in Heilongjiang Province and the study investigated two maize cultivars, JNK728 (Jingnongke 728) and SD5 (Saide 5), under high-density planting conditions (90,000 plants ha^−1^). The treatments were arranged in a factorial design, incorporating four nitrogen levels (0, 120, 240, and 360 kg N ha^−1^) in combination with the presence or absence of a chemical regulator (30% diethyl aminoethyl hexanoate · ethephon), with water serving as the control. Results demonstrated that the integration of 240 kg N ha^−1^ with chemical regulation significantly enhanced photosynthetic capacity—elevating chlorophyll content (SPAD), net photosynthetic rate (Pn), and activities of ribulose-1,5-bisphosphate carboxylase/oxygenase (RuBPCase) and phosphoenolpyruvate carboxylase (PEPCase)—while improving canopy structure through increased leaf area index (LAI) and optimized light distribution. This strategy also reinforced lodging resistance by optimizing plant morphology (reducing plant height and center of gravity), strengthening basal internodes (increasing stem diameter, dry weight per unit length, and mechanical strength), and promoting accumulation of stem structural components (cellulose, hemicellulose, lignin). Additionally, it facilitated post-anthesis nitrogen translocation to grains and up-regulated key nitrogen metabolism enzymes (glutamate synthetase-GS, glutamate dehydrogenase-GDH, and glutamate-pyruvate transaminase-GPT), thereby boosting nitrogen use efficiency. In contrast, excessive nitrogen (360 kg N ha^−1^) suppressed these benefits and increased lodging. Consequently, the combined application of 240 kg N ha^−1^ with chemical regulation achieved the highest yield, proving an effective approach for synergistically enhancing photosynthesis, lodging resistance, and nitrogen utilization in high-density maize systems.

## 1. Introduction

Global population growth and climate change are continuing to exert pressure on global food security [1]. Maize (*Zea mays* L.) is a vital crop for grain, feed, and industrial raw materials, and enhancing maize yield is crucial for securing global supply [2]. Given the persistent decline in arable land resources, increasing planting density represents the most cost-effective approach to fully utilize light and heat resources and maximize the yield potential of maize [3,4]. The significant increase in maize yield over recent decades is widely recognized as being largely due to higher planting densities [5]. A previous study indicated that the optimal planting density for maize in China is 52,500–67,500 plants ha^−1^ [6], while Luo et al. suggested that the maximum maize yield in Northeast China can be achieved at a planting density of 85,000 plants ha^−1^ [7].

However, high planting density intensifies competition among individual plants, leading to a series of negative impacts that ultimately constrain yield gains [8]. The primary issue induced by high-density stress is the deterioration of canopy structure, characterized by an excessively high leaf area index (LAI), a decline in light availability for middle and lower leaves, and the creation of a pronounced shading environment within the canopy [9]. The decline in photosynthesis in the middle and lower canopy layers limits photoassimilate production and accumulation. An insufficient supply of assimilates to developing grains consequently causes yield losses [10]. Competition for nutrients and light among plants adversely affects stem growth. This is evidenced by increased plant and ear height, a higher internode length-to-diameter ratio, and reduced mechanical strength, which consequently significantly increases lodging risk [11]. Lodging is a major cause of crop yield loss under high planting density. Lodging resistance depends on the morphological traits, internal composition, and anatomical structure of the stem. Research indicates that high plant density inhibits the biosynthesis of cellulose, hemicellulose, and lignin in the stem, leading to reduced stem mechanical strength [12,13]. Concurrently, the deterioration of anatomical structures, such as a reduced number of vascular bundles and thinner mechanical tissue layers, further compromises resistance to stem bending [14].

Nitrogen fertilizer serves as a significant measure for mitigating the detrimental effects induced by high planting density. Rational nitrogen supply enhances maize photosynthesis, thereby increasing the accumulation of assimilates and improving grain yield [15]. Studies indicate that optimal nitrogen rates can significantly enhance grain yield and dry matter accumulation under high planting density [16]. However, excessive nitrogen application promotes overly vigorous leaf growth, which deteriorates light conditions within the canopy and is detrimental to biomass and yield formation [4]. Consequently, under high planting density, merely relying on nitrogen fertilizer may fail to raise yields. Moreover, it can exacerbate excessive vegetative growth, increase lodging risk, and aggravate fertilizer waste [17]. Chemical regulation is an effective strategy for reducing lodging risk in maize production. Plant growth regulators (PGRs) can inhibit internode elongation by modulating endogenous hormone levels, thereby enhancing lodging resistance [18]. Studies have shown that PGRs effectively reduced plant height and ear height, increased stem diameter, and consequently improved lodging resistance [19]. Furthermore, chemical regulation is effective in modulating maize canopy architecture. Studies have shown that it can adjust leaf size and spatial distribution, thereby improving canopy structure and internal light conditions, which creates a more favorable environment for photosynthesis [20]. The plant growth regulator used in this study was 30% diethyl aminoethyl hexanoate · ethephon. Diethyl aminoethyl hexanoate could improve photosynthetic capacity in leaves and increase maize yield, while ethephon could reduce internode length, enhance cellulose and lignin content in internodes, and strengthen stem lodging resistance. However, improper management of chemical regulation may lead to excessive suppression of vegetative growth, which can compromise photosynthetic capacity and consequently reduce grain yield [21]. Hence, achieving an optimal balance between lodging resistance and high yield under high planting density is of significant importance in agricultural production. While current research has predominantly centered on the separate impacts of either nitrogen fertilizer or chemical regulation on maize production, the synergistic role of combined nitrogen management and chemical regulation in reconciling the conflict between lodging resistance and high yield under high planting density remains unclear. Therefore, elucidating the regulating mechanisms of nitrogen and chemical regulation on maize growth is crucial for advancing high-yield, high-efficiency, and lodging-resistant maize cultivation.

Therefore, this study established a field experiment with different nitrogen application rates and chemical regulation treatments under high planting density. The objectives were to investigate: (1) the regulatory effects of nitrogen and chemical regulation on leaf photosynthetic capacity and canopy structure; (2) their impacts on stem lodging resistance, elucidating the underlying mechanisms through morphology, mechanical strength, biochemical composition, and anatomical structure; (3) their influences on plant nitrogen uptake and utilization; and (4) their effects on grain yield. We hypothesized that combining nitrogen fertilizer with chemical regulation would improve maize yield and nitrogen use efficiency through the coordinated enhancement of canopy photosynthesis and stem strength. The results will provide valuable insights for developing sustainable maize cultivation.

## 2. Results

### 2.1. Photosynthetic Characteristics

#### 2.1.1. Chlorophyll Content (SPAD Value), Net Photosynthetic Rate (Pn), and Maximum Quantum Yield (Fv/Fm)

**Figure 1:**
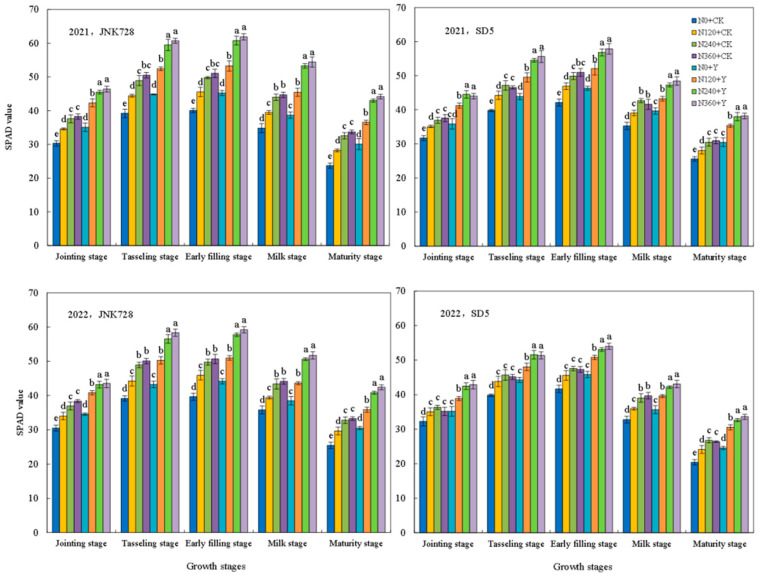
Effects of nitrogen fertilizer and chemical regulation on the chlorophyll content (SPAD) value of maize leaves in 2021 and 2022. Columns within the same growth stage marked with different letters indicate statistically significant differences at *p* < 0.05. Vertical bars mean ± SE. JNK728 and SD5 indicate maize varieties Jingnongke 728 and Saide 5, respectively. N0 + CK, N120 + CK, N240 + CK, and N360 + CK represent nitrogen application rates of 0, 120, 240, and 360 kg ha^−1^ combined with water application, while N0 + Y, N120 + Y, N240 + Y, and N360 + Y represent the nitrogen application rates of 0, 120, 240, and 360 kg ha^−1^ combined with plant growth regulator.

**Figure 2:**
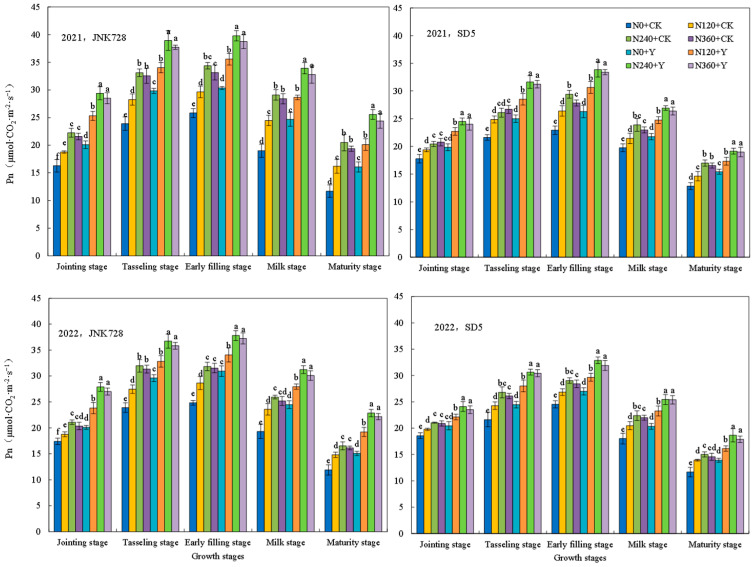
Effects of nitrogen fertilizer and chemical regulation on the net photosynthetic rate (Pn) of maize leaves in 2021 and 2022. Columns within the same growth stage marked with different letters indicate statistically significant differences at *p* < 0.05. Vertical bars mean ± SE. JNK728 and SD5 indicate maize varieties Jingnongke 728 and Saide 5, respectively. N0 + CK, N120 + CK, N240 + CK, and N360 + CK represent nitrogen application rates of 0, 120, 240, and 360 kg ha^−1^ combined with water application, while N0 + Y, N120 + Y, N240 + Y, and N360 + Y represent the nitrogen application rates of 0, 120, 240, and 360 kg ha^−1^ combined with plant growth regulator.

**Figure 3:**
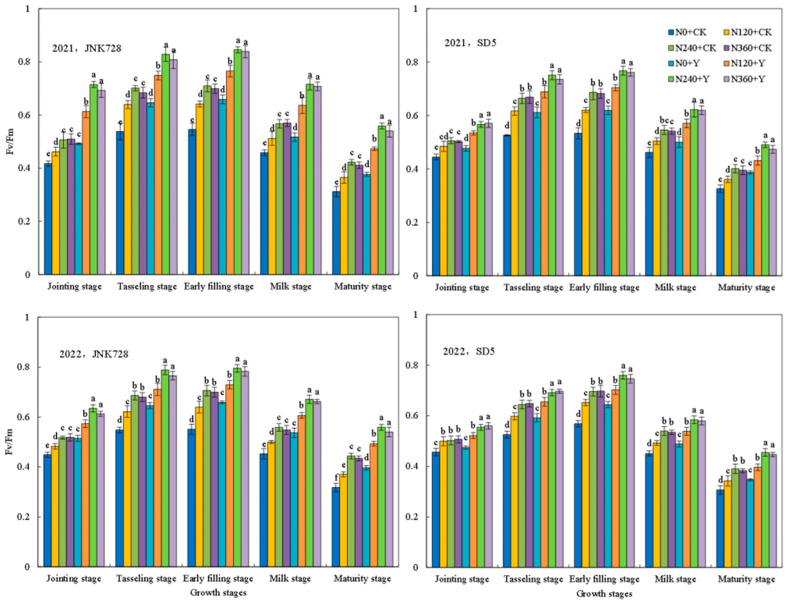
Effects of nitrogen fertilizer and chemical regulation on the maximum quantum yield (Fv/Fm) of maize leaves in 2021 and 2022. Columns within the same growth stage marked with different letters indicate statistically significant differences at *p* < 0.05. Vertical bars mean ± SE. JNK728 and SD5 indicate maize varieties Jingnongke 728 and Saide 5, respectively. N0 + CK, N120 + CK, N240 + CK, and N360 + CK represent nitrogen application rates of 0, 120, 240, and 360 kg ha^−1^ combined with water application, while N0 + Y, N120 + Y, N240 + Y, and N360 + Y represent the nitrogen application rates of 0, 120, 240, and 360 kg ha^−1^ combined with plant growth regulator.

Photosynthetic characteristics are critical determinants of maize yield. The SPAD value, Pn, and Fv/Fm exhibited consistent variation trends across two growing seasons (Figure 1, Figure 2 and Figure 3). All parameters increased from the jointing stage, reached their peak at the early filling stage, and subsequently declined. Nitrogen fertilizer and chemical regulation significantly improved maize photosynthetic performance under high planting density. Compared with 0 kg N ha^−1^ (N0), the application of 240 kg N ha^−1^ (N240) significantly enhanced photosynthetic indexes at all growth stages. At the early filling stage, the N240 treatment increased SPAD, Pn, and Fv/Fm by an average of 28.9%, 28.5%, and 26.1% in JNK728, and by 18.0%, 24.3%, and 23.0% in SD5, respectively. However, there are no significant differences between N240 and 360 kg N ha^−1^ (N360) treatments. Compared with water application (CK), chemical regulation significantly increased SPAD, Pn, and Fv/Fm by 16.3%, 18.6%, and 17.3% in JNK728, and by 11.8%, 14.0%, and 11.1% in SD5, respectively.

#### 2.1.2. Ribulose-1,5-bisphosphate Carboxylase/Oxygenase (RuBPCase) and Phosphoenolpyruvate Carboxylase (PEPCase) Activities

**Figure 4:**
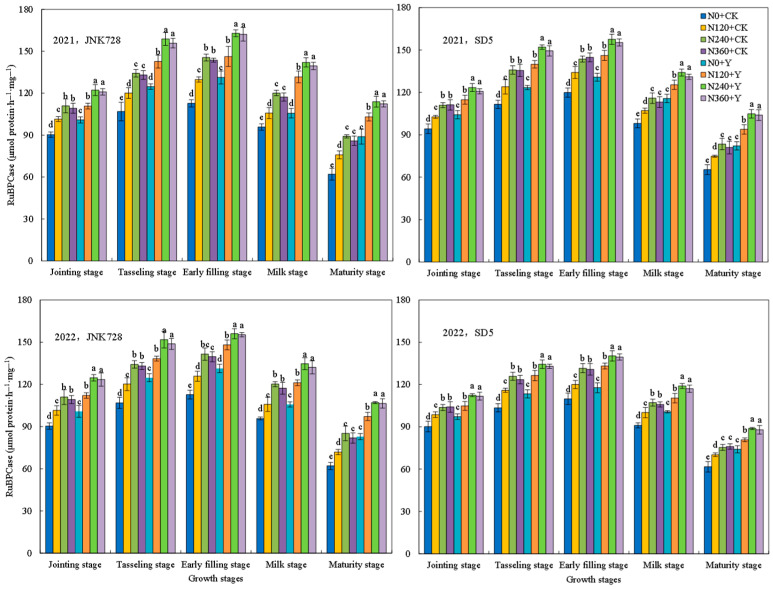
Effects of nitrogen fertilizer and chemical regulation on the ribulose-1,5-bisphosphate carboxylase/oxygenase (RuBPCase) activity of maize leaves in 2021 and 2022. Columns within the same growth stage marked with different letters indicate statistically significant differences at *p* < 0.05. Vertical bars mean ± SE. JNK728 and SD5 indicate maize varieties Jingnongke 728 and Saide 5, respectively. N0 + CK, N120 + CK, N240 + CK, and N360 + CK represent nitrogen application rates of 0, 120, 240, and 360 kg ha^−1^ combined with water application, while N0 + Y, N120 + Y, N240 + Y, and N360 + Y represent the nitrogen application rates of 0, 120, 240, and 360 kg ha^−1^ combined with plant growth regulator.

**Figure 5:**
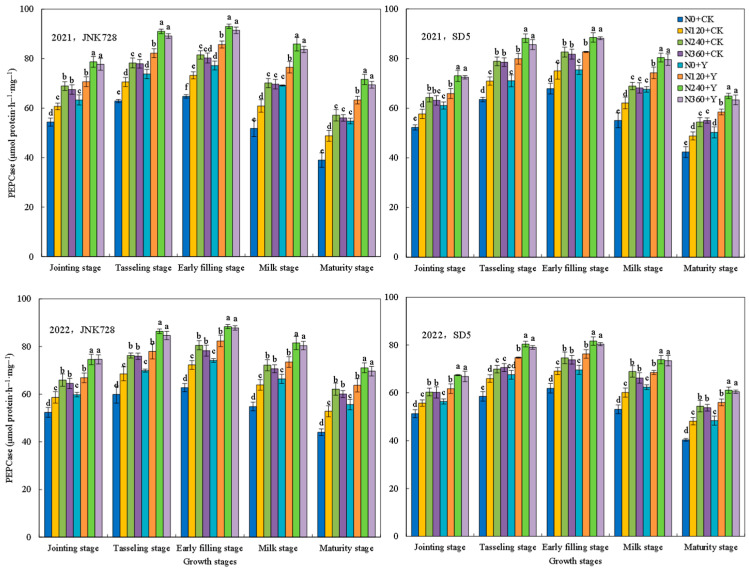
Effects of nitrogen fertilizer and chemical regulation on the phosphoenolpyruvate carboxylase (PEPCase) activity of maize leaves in 2021 and 2022. Columns within the same growth stage marked with different letters indicate statistically significant differences at *p* < 0.05. Vertical bars mean ± SE. JNK728 and SD5 indicate maize varieties Jingnongke 728 and Saide 5, respectively. N0 + CK, N120 + CK, N240 + CK, and N360 + CK represent nitrogen application rates of 0, 120, 240, and 360 kg ha^−1^ combined with water application, while N0 + Y, N120 + Y, N240 + Y, and N360 + Y represent the nitrogen application rates of 0, 120, 240, and 360 kg ha^−1^ combined with plant growth regulator.

RuBPCase and PEPCase are key enzymes in photosynthetic carbon assimilation in maize. The two enzymes’ activities increased consistently from the jointing stages, peaked at the early filling stage, and subsequently declined (Figure 4 and Figure 5). Nitrogen fertilizer significantly increased RuBPCase and PEPCase activities, while no significant difference was observed between the N240 and N360 treatments. Chemical regulation also increased enzyme activities, with RuBPCase and PEPCase activities elevated by 13.5% and 14.6% in JNK728, and 8.3% and 9.5% in SD5, respectively. These results indicate that nitrogen fertilizer and chemical regulation are effective measures for sustaining photosynthetic capacity in high-density maize cropping systems.

#### 2.1.3. Leaf Area Index (LAI) and Canopy Light Transmittance

**Figure 6:**
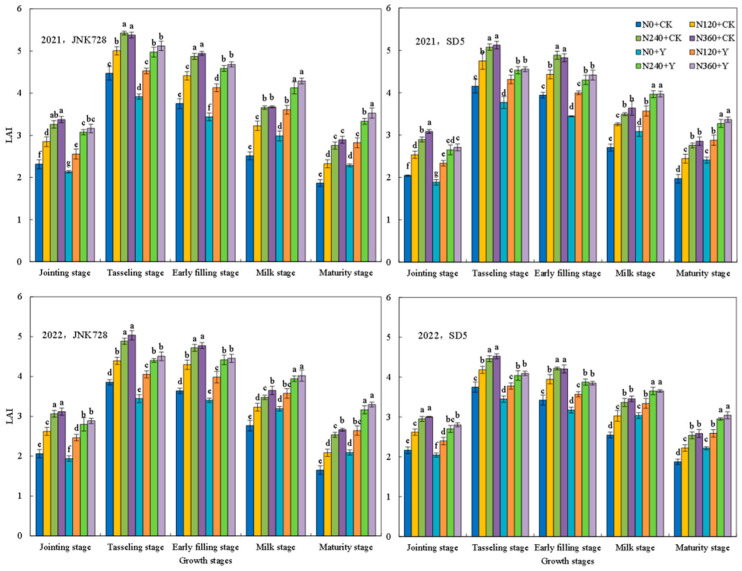
Effects of nitrogen fertilizer and chemical regulation on leaf area index (LAI) in 2021 and 2022. Columns within the same growth stage marked with different letters indicate statistically significant differences at *p* < 0.05. Vertical bars mean ± SE. JNK728 and SD5 indicate maize varieties Jingnongke 728 and Saide 5, respectively. N0 + CK, N120 + CK, N240 + CK, and N360 + CK represent nitrogen application rates of 0, 120, 240, and 360 kg ha^−1^ combined with water application, while N0 + Y, N120 + Y, N240 + Y, and N360 + Y represent the nitrogen application rates of 0, 120, 240, and 360 kg ha^−1^ combined with plant growth regulator.

LAI is a key index of canopy structure and photosynthetic area, reflecting light interception and utilization of the population. The LAI increased consistently from the jointing stages, peaked at tasseling, and subsequently declined (Figure 6). Nitrogen application significantly enhanced the LAI at each growth stage, while no significant difference was observed between the N240 and N360 treatments. Chemical regulation significantly reduced the LAI during the jointing stage to the early filling stage, while it increased LAI during the milk stage to the maturity stage.

**Figure 7:**
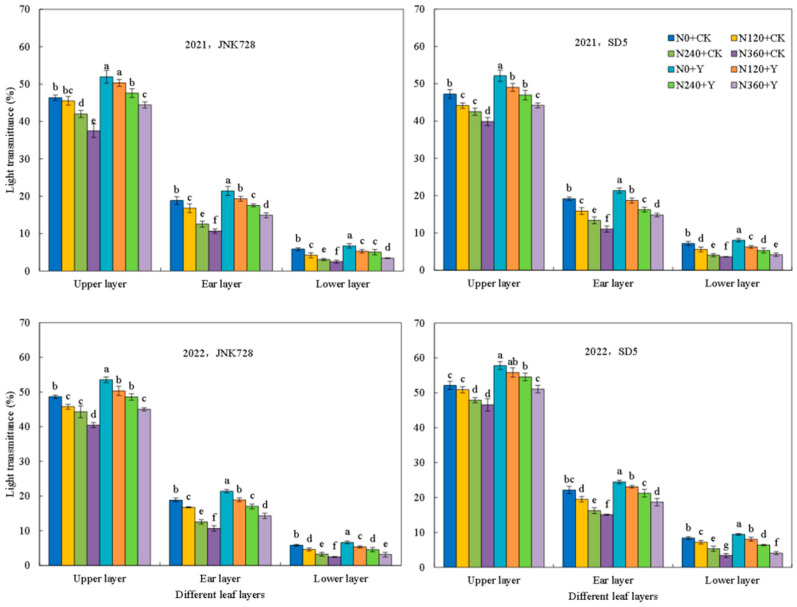
Effects of nitrogen fertilizer and chemical regulation on the light transmittance of the canopy in 2021 and 2022. Columns within the same growth stage marked with different letters indicate statistically significant differences at *p* < 0.05. Vertical bars mean ± SE. JNK728 and SD5 indicate maize varieties Jingnongke 728 and Saide 5, respectively. N0 + CK, N120 + CK, N240 + CK, and N360 + CK represent nitrogen application rates of 0, 120, 240, and 360 kg ha^−1^ combined with water application, while N0 + Y, N120 + Y, N240 + Y, and N360 + Y represent the nitrogen application rates of 0, 120, 240, and 360 kg ha^−1^ combined with plant growth regulator.

At the early filling stage, light transmittance within the canopy followed the pattern: upper layer > ear layer > lower layer (Figure 7). Nitrogen application significantly reduced light transmittance across all canopy layers. Chemical regulation significantly increased light transmittance for JNK728 and SD5, with corresponding increases of 11.8% and 10.9% in the upper layer, 23.1% and 19.7% in the ear layer, and 26.7% and 15.8% in the lower layer, respectively. Thus, the nitrogen fertilizer and chemical regulation improved canopy light transmittance and optimized population photosynthetic performance.

### 2.2. Lodging Resistance Characteristics

#### 2.2.1. Plant Morphology

**Figure 8:**
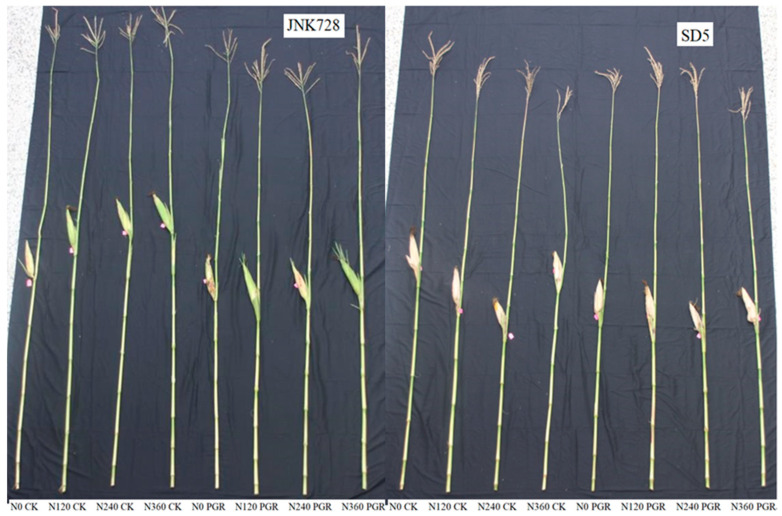
Effects of nitrogen fertilizer and chemical regulation on plant morphology. JNK728 and SD5 indicate maize varieties Jingnongke 728 and Saide 5, respectively. N0 CK, N120 CK, N240 CK, and N360 CK represent nitrogen application rates of 0, 120, 240, and 360 kg ha^−1^ combined with water application, while N0 PGR, N120 PGR, N240 PGR, and N360 PGR represent the nitrogen application rates of 0, 120, 240, and 360 kg ha^−1^ combined with plant growth regulator.

Plant height, ear height, ear height coefficient, and center of gravity height are key morphological traits in maize, significantly influencing grain yield and lodging resistance. Nitrogen fertilizer and chemical regulation significantly affected these traits at the milk stage under high planting density (Figure 8 and Table 1). Nitrogen fertilizer significantly influenced plant morphological parameters. Compared to the N0 treatment, N120, N240, and N360 treatments increased plant height by 1.1%, 2.6%, and 5.7%; ear height by 3.2%, 8.7%, and 11.8%; ear height coefficient by 2.0%, 4.6%, and 5.7%; and center of gravity height by 3.2%, 6.2%, and 12.0%, respectively. In contrast, chemical regulation significantly reduced plant height, ear height, ear height coefficient, and center of gravity height by 5.2%, 10.6%, 5.1%, and 9.5%, respectively, compared to the water application treatment (CK). These indicated that nitrogen input potentially increases lodging risk, whereas chemical regulation effectively optimizes plant morphology and contributes to improved lodging resistance.

#### 2.2.2. Internode Morphology

The third basal internode of maize is critically associated with lodging resistance, serving as a supported part that is particularly susceptible to bending or breaking. Nitrogen fertilizer and chemical regulation significantly influenced the third basal internode morphology at the early filling stage (Table 2). Nitrogen fertilizer significantly increased internode length and stem diameter. Compared with N0 treatment, the N120, N240, and N360 treatments increased internode length by 2.7%, 5.6%, and 12.9%, and stem diameter by 7.2%, 14.8%, and 13.8%, respectively, while there was no significant difference in stem diameter between the N240 and N360 treatments. Furthermore, chemical regulation significantly reduced internode length while increasing stem diameter compared to CK. The dry weight per unit length reflected stem plumpness, and is closely related to stem mechanical strength. With increasing nitrogen application rate, the dry weight per unit length significantly increased; however, excessively high nitrogen levels led to its reduction. Chemical regulation significantly increased the dry weight per unit length. These indicated that under high planting density, inappropriate nitrogen application can lead to reduced stem plumpness, while chemical regulation can decrease basal internode length, increase stem diameter, and improve stem plumpness.

#### 2.2.3. Bending Strength and Rind Penetration Strength

**Figure 9:**
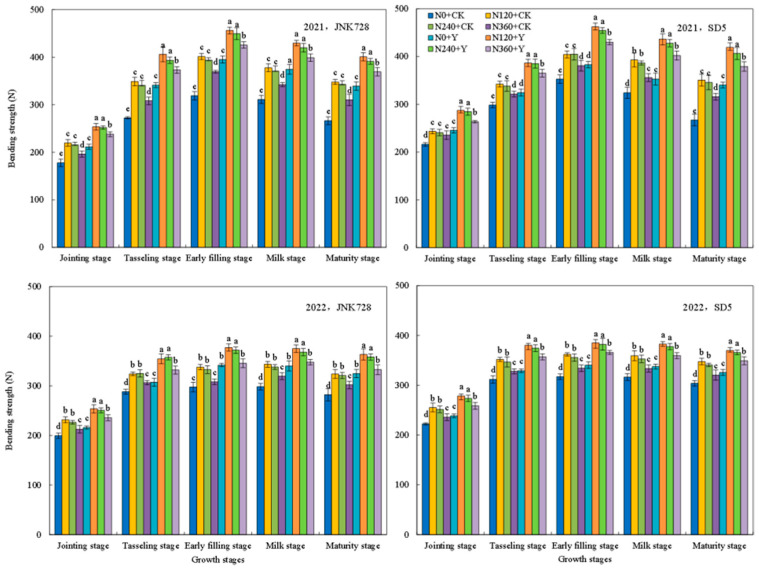
Effects of nitrogen fertilizer and chemical regulation on the bending strength of the third basal internode in 2021 and 2022. Columns within the same growth stage marked with different letters indicate statistically significant differences at *p* < 0.05. Vertical bars mean ± SE. JNK728 and SD5 indicate maize varieties Jingnongke 728 and Saide 5, respectively. N0 + CK, N120 + CK, N240 + CK, and N360 + CK represent nitrogen application rates of 0, 120, 240, and 360 kg ha^−1^ combined with water application, while N0 + Y, N120 + Y, N240 + Y, and N360 + Y represent the nitrogen application rates of 0, 120, 240, and 360 kg ha^−1^ combined with plant growth regulator.

**Figure 10:**
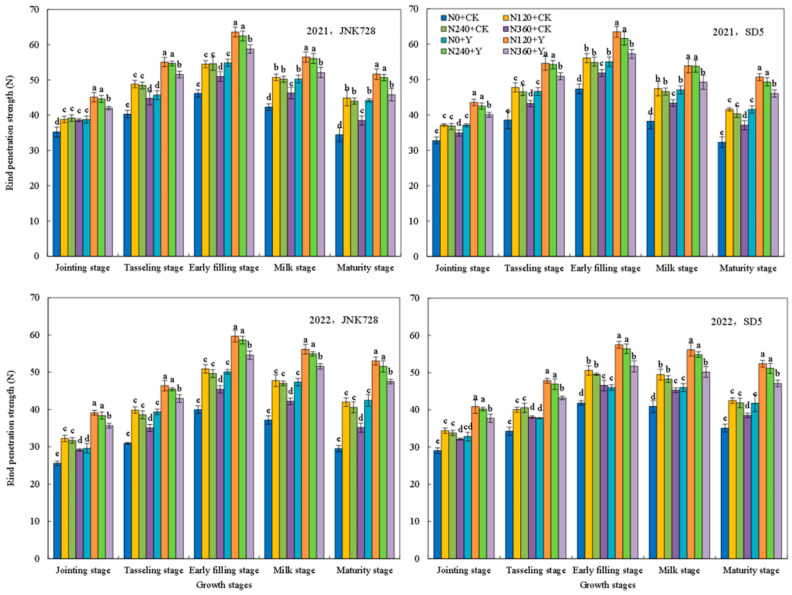
Effects of nitrogen fertilizer and chemical regulation on the rind penetration strength of the third basal internode in 2021 and 2022. Columns within the same growth stage marked with different letters indicate statistically significant differences at *p* < 0.05. Vertical bars mean ± SE. JNK728 and SD5 indicate maize varieties Jingnongke 728 and Saide 5, respectively. N0 + CK, N120 + CK, N240 + CK, and N360 + CK represent nitrogen application rates of 0, 120, 240, and 360 kg ha^−1^ combined with water application, while N0 + Y, N120 + Y, N240 + Y, and N360 + Y represent the nitrogen application rates of 0, 120, 240, and 360 kg ha^−1^ combined with plant growth regulator.

The bending strength and rind penetration strength of the third basal internode are key indicators of stem mechanical strength. During the maize growth period, two parameters initially increased, peaked at the early filling stage (Figure 9 and Figure 10), and subsequently decreased. Bending strength increased initially and then declined with increasing nitrogen application rate. At the early filling stage, compared with N0, the bending strength increased under N120, N240, and N360 treatments by 16.0%, 14.5%, and 7.7%, respectively. A similar trend was observed for rind penetration strength. Consequently, the N120 and N240 treatments resulted in higher bending strength and rind penetration strength, whereas an excessive nitrogen application (N360) led to decreases. Chemical regulation further enhanced these mechanical characteristics. Compared with CK, chemical regulation increased the bending strength and rind penetration strength by 14.6% and 10.0% for JNK728, and by 18.0% and 12.6% for SD5, respectively, at the early filling stage. In conclusion, appropriate nitrogen fertilization combined with chemical regulation significantly enhanced stem mechanical strength under high planting density.

#### 2.2.4. Lodging Resistance Index

**Figure 11:**
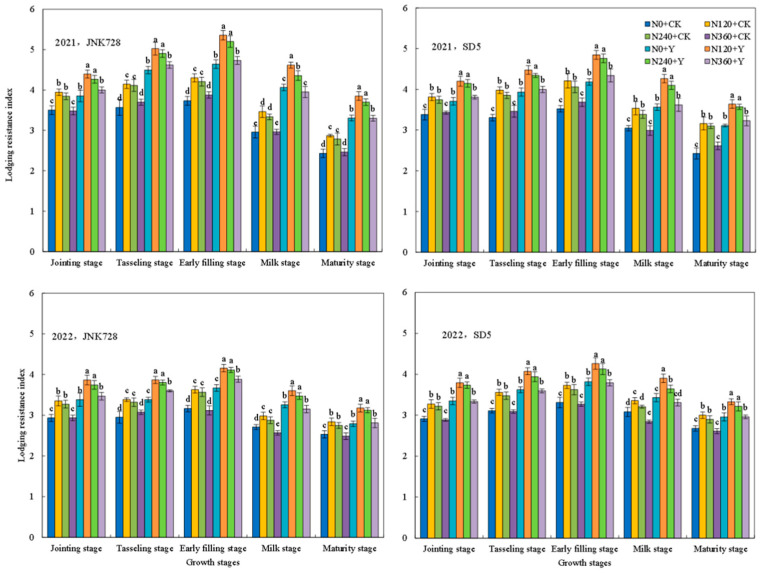
Effects of nitrogen fertilizer and chemical regulation on the lodging resistance index in 2021 and 2022. Columns within the same growth stage marked with different letters indicate statistically significant differences at *p* < 0.05. Vertical bars mean ± SE. JNK728 and SD5 indicate maize varieties Jingnongke 728 and Saide 5, respectively. N0 + CK, N120 + CK, N240 + CK, and N360 + CK represent nitrogen application rates of 0, 120, 240, and 360 kg ha^−1^ combined with water application, while N0 + Y, N120 + Y, N240 + Y, and N360 + Y represent the nitrogen application rates of 0, 120, 240, and 360 kg ha^−1^ combined with plant growth regulator.

During the maize growing season, the stem lodging resistance index initially increased, reached a maximum at the early filling stage, and subsequently decreased (Figure 11). Nitrogen fertilizer and chemical regulation significantly affected the lodging resistance index of maize under high planting density. With the nitrogen rate increased, the lodging resistance index first increased and then decreased. Specifically, the lodging resistance indices were relatively high under the nitrogen application rates of N120 and N240, while an excessively high nitrogen application (N360) conversely led to a decrease in the lodging resistance index. Chemical regulation could enhance the lodging resistance index. Compared with CK, the lodging resistance indices of JNK728 and SD5 increased by 20.8% and 16.0%, respectively, at the early filling stage. This indicated that appropriate nitrogen application and chemical regulation can improve the lodging resistance of maize under high planting density.

#### 2.2.5. Lodging Rate

**Figure 12:**
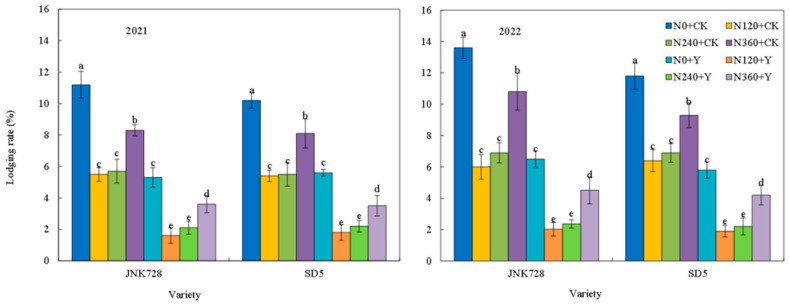
Effects of nitrogen fertilizer and chemical regulation on lodging rate in 2021 and 2022. Columns within the same variety marked with different letters indicate statistically significant differences at *p* < 0.05. Vertical bars mean ± SE. JNK728 and SD5 indicate maize varieties Jingnongke 728 and Saide 5, respectively. N0 + CK, N120 + CK, N240 + CK, and N360 + CK represent nitrogen application rates of 0, 120, 240, and 360 kg ha^−1^ combined with water application, while N0 + Y, N120 + Y, N240 + Y, and N360 + Y represent the nitrogen application rates of 0, 120, 240, and 360 kg ha^−1^ combined with plant growth regulator.

Nitrogen fertilizer and chemical regulation significantly affected the lodging rate of maize at the maturity stage under high planting density (Figure 12). With the increase in nitrogen application rate, the lodging rate first decreased and then increased. Specifically, the lodging rates under the N120 and N240 treatments were relatively low, showing reductions of 50.2% and 46.6%, respectively, compared to the N0 treatment. Chemical regulation could further reduce the lodging rate under different nitrogen application levels. This indicated that appropriate nitrogen fertilizer and chemical regulation can significantly reduce the lodging rate under high planting density.

#### 2.2.6. Soluble Sugar, Cellulose, Hemicellulose, and Lignin Contents

**Figure 13:**
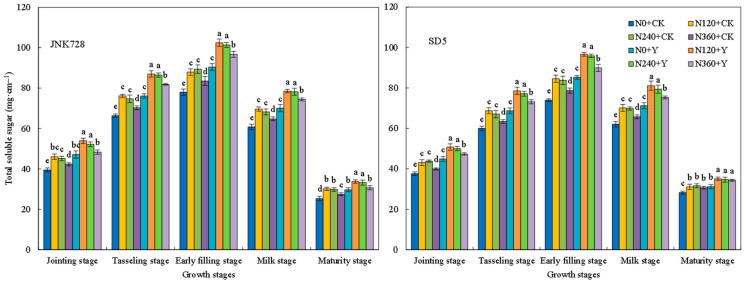
Effects of nitrogen fertilizer and chemical regulation on the soluble sugar of the stem in 2021. Columns within the same growth stage marked with different letters indicate statistically significant differences at *p* < 0.05. Vertical bars mean ± SE. JNK728 and SD5 indicate maize varieties Jingnongke 728 and Saide 5, respectively. N0 + CK, N120 + CK, N240 + CK, and N360 + CK represent nitrogen application rates of 0, 120, 240, and 360 kg ha^−1^ combined with water application, while N0 + Y, N120 + Y, N240 + Y, and N360 + Y represent the nitrogen application rates of 0, 120, 240, and 360 kg ha^−1^ combined with plant growth regulator.

**Figure 14:**
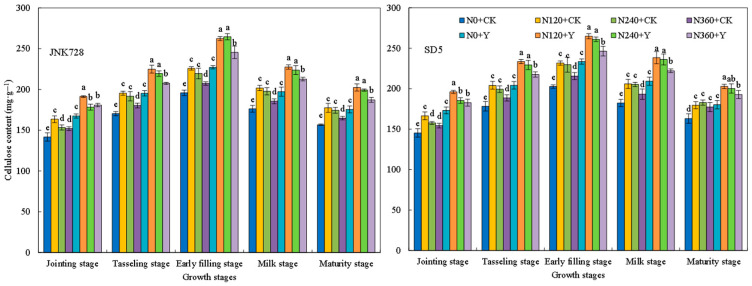
Effects of nitrogen fertilizer and chemical regulation on the cellulose of the stem in 2021. Columns within the same growth stage marked with different letters indicate statistically significant differences at *p* < 0.05. Vertical bars mean ± SE. JNK728 and SD5 indicate maize varieties Jingnongke 728 and Saide 5, respectively. N0 + CK, N120 + CK, N240 + CK, and N360 + CK represent nitrogen application rates of 0, 120, 240, and 360 kg ha^−1^ combined with water application, while N0 + Y, N120 + Y, N240 + Y, and N360 + Y represent the nitrogen application rates of 0, 120, 240, and 360 kg ha^−1^ combined with plant growth regulator.

**Figure 15:**
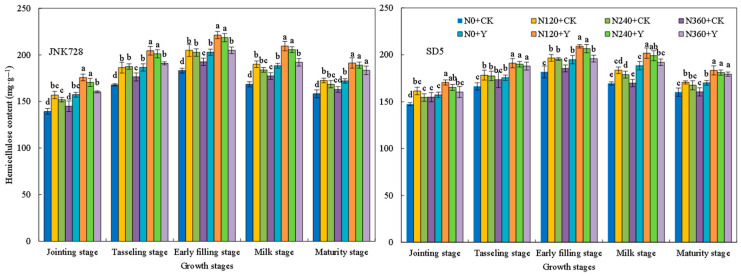
Effects of nitrogen fertilizer and chemical regulation on the hemicellulose of the stem in 2021. Columns within the same growth stage marked with different letters indicate statistically significant differences at *p* < 0.05. Vertical bars mean ± SE. JNK728 and SD5 indicate maize varieties Jingnongke 728 and Saide 5, respectively. N0 + CK, N120 + CK, N240 + CK, and N360 + CK represent nitrogen application rates of 0, 120, 240, and 360 kg ha^−1^ combined with water application, while N0 + Y, N120 + Y, N240 + Y, and N360 + Y represent the nitrogen application rates of 0, 120, 240, and 360 kg ha^−1^ combined with plant growth regulator.

**Figure 16:**
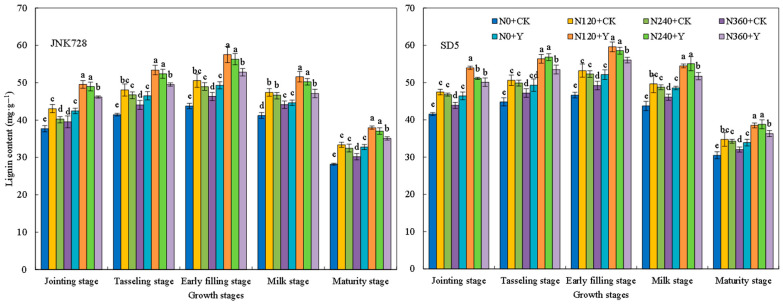
Effects of nitrogen fertilizer and chemical regulation on the lignin of the stem in 2021. Columns within the same growth stage marked with different letters indicate statistically significant differences at *p* < 0.05. Vertical bars mean ± SE. JNK728 and SD5 indicate maize varieties Jingnongke 728 and Saide 5, respectively. N0 + CK, N120 + CK, N240 + CK, and N360 + CK represent nitrogen application rates of 0, 120, 240, and 360 kg ha^−1^ combined with water application, while N0 + Y, N120 + Y, N240 + Y, and N360 + Y represent the nitrogen application rates of 0, 120, 240, and 360 kg ha^−1^ combined with plant growth regulator.

Soluble sugars, cellulose, hemicellulose, and lignin are important storage substances that affect the mechanical strength of the stem. During the growth period, the storage substances in the internodes first increased and then decreased, reaching a peak at the early filling stage (Figure 13, Figure 14, Figure 15 and Figure 16). With the increase in nitrogen rate, the contents of soluble sugars, cellulose, hemicellulose, and lignin in internodes first increased and then decreased. Specifically, the storage substances contents in internodes were relatively high under the N120 and N240 treatments, while an excessively high nitrogen application rate (N360 treatment) led to a decrease in these contents. Chemical regulation could further increase the contents of soluble sugars, cellulose, hemicellulose, and lignin in internodes by 15.0%, 16.1%, 7.2%, 13.9%, and 13.2%, respectively, compared with CK (with the early filling stage taken as an example). This indicated that appropriate nitrogen fertilizer and chemical regulation can significantly increase the content of storage substances in internodes under high planting density.

#### 2.2.7. Correlation Between Stem Mechanical Traits and Internode Carbohydrates Contents

The carbohydrates contents of the internode were significantly correlated with stem mechanical characteristics (Table 3). The contents of soluble sugar, cellulose, hemicellulose and lignin in stem exhibited significant positive correlation with stem bending strength, rind penetration strength and lodging resistance index, and significant negative correlation with lodging rate. These results indicate that the accumulated carbohydrates in the internodes are closely associated with the stalk mechanical properties and lodging rate, and favorable internode quality is conducive to the improvement of lodging resistance.

#### 2.2.8. Lignin-Related Enzymes

**Figure 17:**
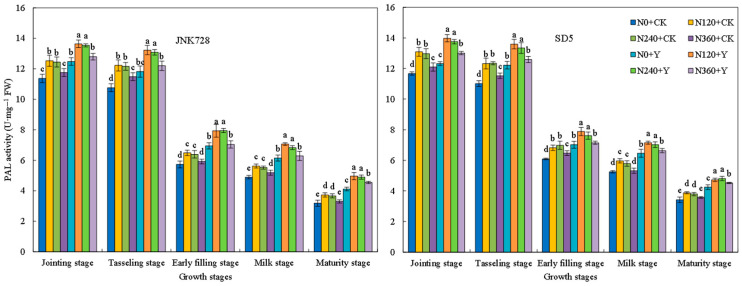
Effects of nitrogen fertilizer and chemical regulation on the phenylalanine ammonia-lyase (PAL) activity of the stem in 2021. Columns within the same growth stage marked with different letters indicate statistically significant differences at *p* < 0.05. Vertical bars mean ± SE. JNK728 and SD5 indicate maize varieties Jingnongke 728 and Saide 5, respectively. N0 + CK, N120 + CK, N240 + CK, and N360 + CK represent nitrogen application rates of 0, 120, 240, and 360 kg ha^−1^ combined with water application, while N0 + Y, N120 + Y, N240 + Y, and N360 + Y represent the nitrogen application rates of 0, 120, 240, and 360 kg ha^−1^ combined with plant growth regulator.

**Figure 18:**
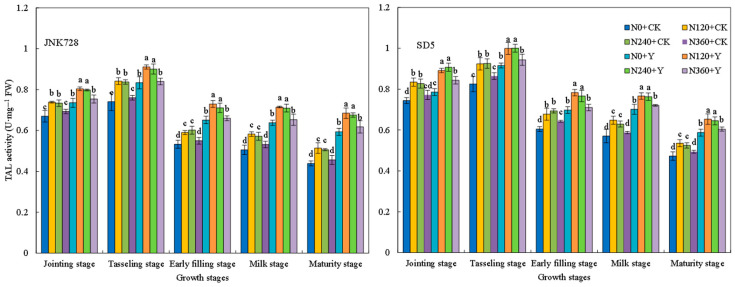
Effects of nitrogen fertilizer and chemical regulation on the tyrosine ammonia-lyase (TAL) activity of the stem in 2021. Columns within the same growth stage marked with different letters indicate statistically significant differences at *p* < 0.05. Vertical bars mean ± SE. JNK728 and SD5 indicate maize varieties Jingnongke 728 and Saide 5, respectively. N0 + CK, N120 + CK, N240 + CK, and N360 + CK represent nitrogen application rates of 0, 120, 240, and 360 kg ha^−1^ combined with water application, while N0 + Y, N120 + Y, N240 + Y, and N360 + Y represent the nitrogen application rates of 0, 120, 240, and 360 kg ha^−1^ combined with plant growth regulator.

**Figure 19:**
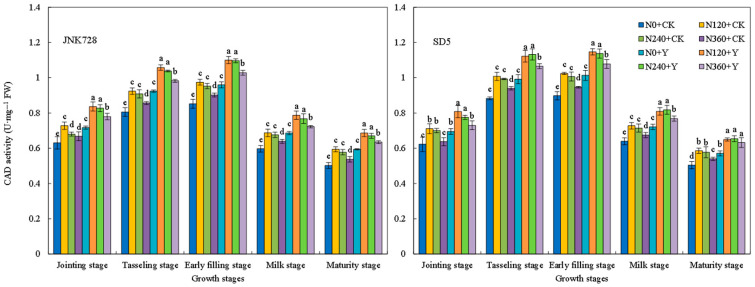
Effects of nitrogen fertilizer and chemical regulation on the cinnamyl alcohol dehydrogenase (CAD) activity of the stem in 2021. Columns within the same growth stage marked with different letters indicate statistically significant differences at *p* < 0.05. Vertical bars mean ± SE. JNK728 and SD5 indicate maize varieties Jingnongke 728 and Saide 5, respectively. N0 + CK, N120 + CK, N240 + CK, and N360 + CK represent nitrogen application rates of 0, 120, 240, and 360 kg ha^−1^ combined with water application, while N0 + Y, N120 + Y, N240 + Y, and N360 + Y represent the nitrogen application rates of 0, 120, 240, and 360 kg ha^−1^ combined with plant growth regulator.

**Figure 20:**
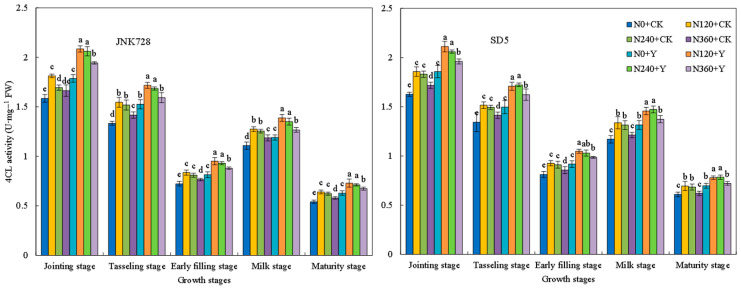
Effects of nitrogen fertilizer and chemical regulation on the 4-coumarate:CoA ligase (4CL) activity of the stem in 2021. Columns within the same growth stage marked with different letters indicate statistically significant differences at *p* < 0.05. Vertical bars mean ± SE. JNK728 and SD5 indicate maize varieties Jingnongke 728 and Saide 5, respectively. N0 + CK, N120 + CK, N240 + CK, and N360 + CK represent nitrogen application rates of 0, 120, 240, and 360 kg ha^−1^ combined with water application, while N0 + Y, N120 + Y, N240 + Y, and N360 + Y represent the nitrogen application rates of 0, 120, 240, and 360 kg ha^−1^ combined with plant growth regulator.

Phenylalanine ammonia-lyase (PAL) activity exhibited a decreasing trend during growth, with high activity from the jointing to tasseling stages (Figure 17). Tyrosine ammonia-lyase (TAL) activity followed a single-peak curve, peaking at the tasseling stage (Figure 18). Cinnamyl alcohol dehydrogenase (CAD) activity first increased and then decreased, reaching its maximum at the early filling stage (Figure 19). 4-coumarate:CoA ligase (4CL) activity generally decreased, with a slight increase at the milk stage (Figure 20). Nitrogen application (N120–N240) enhanced the activities of these four enzymes but inhibited them at excessive rates (N360) under high planting density. Additionally, chemical regulation improved the activities of all four enzymes across growth stages. Correlation analysis revealed that the internode lignin content was significantly positively correlated with the activities of lignin biosynthesis-related enzymes. Therefore, the increase in lignin-related enzymes activities contribute to higher lignin content. These results indicated that appropriate nitrogen application combined with chemical regulation can effectively enhance the activities of key enzymes involved in lignin synthesis in the third basal internode of maize under high planting density, thereby promoting lignin accumulation.

#### 2.2.9. Anatomical Structure

**Figure 21:**
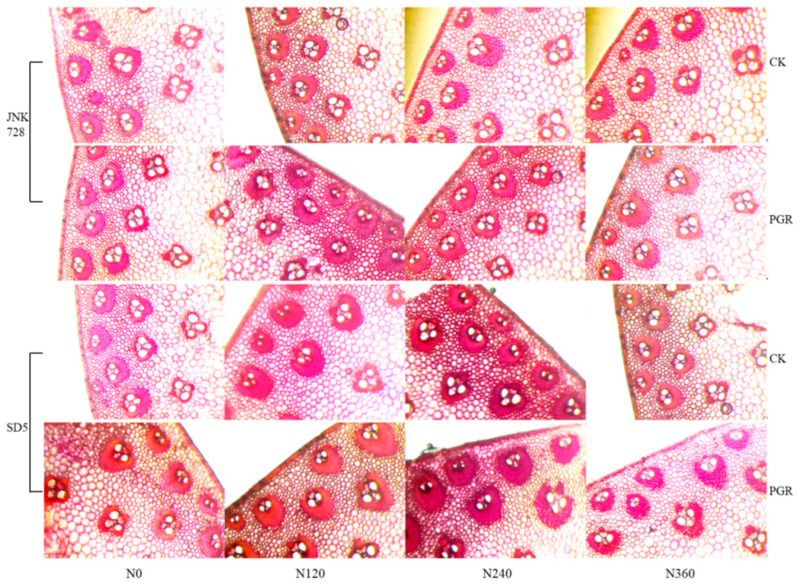
Effects of nitrogen fertilizer and chemical regulation on the anatomical structure of the stem in 2021. JNK728 and SD5 indicate maize varieties Jingnongke 728 and Saide 5, respectively. N0, N120, N240, and N360 represent nitrogen application rates of 0, 120, 240, and 360 kg ha^−1^, respectively. Y and CK represent the application of plant growth regulator and water, respectively.

Stem mechanical strength is closely associated with its anatomical structure. Under high planting density, the third basal internode of maize exhibited larger and more densely arranged vascular bundles under N120 and N240 treatments, whereas smaller, disordered, and uneven-sized vascular bundles were observed under N0 and N360 (Figure 21). Additionally, chemical regulation significantly increased the vascular bundle area.

Nitrogen fertilizer and chemical regulation significantly affected stem anatomical structure at the early filling stage under high planting density. With increasing N rate, cortex thickness, mechanical thickness, mechanical cell layers, and large/small vascular bundles number first increased and then decreased, peaking at N120 and N240, while excessive N (N360) significantly reduced these indices (Table 4). Compared to N0, cortex thickness increased by 21.0%, 17.9%, and 10.7% under N120, N240, and N360, respectively; mechanical thickness by 24.1%, 21.1%, and 12.2%; and mechanical cell layers by 25.5%, 22.6%, and 13.1%. Large/small vascular bundle area increased with N application but decreased under N360. Chemical regulation could increase cortex thickness, mechanical thickness, mechanical cell layers, and large/small vascular bundle number/area by 23.6%, 17.2%, 17.9%, and 8.1%/16.9%, 13.7%/17.2%, respectively, compared to CK. These results indicated that appropriate N application combined with chemical regulation optimized the stem anatomical structure of maize under high planting density, thereby enhancing stem mechanical strength and lodging resistance.

### 2.3. Nitrogen Utilization Characteristics

#### 2.3.1. Plant Nitrogen Accumulation

**Figure 22:**
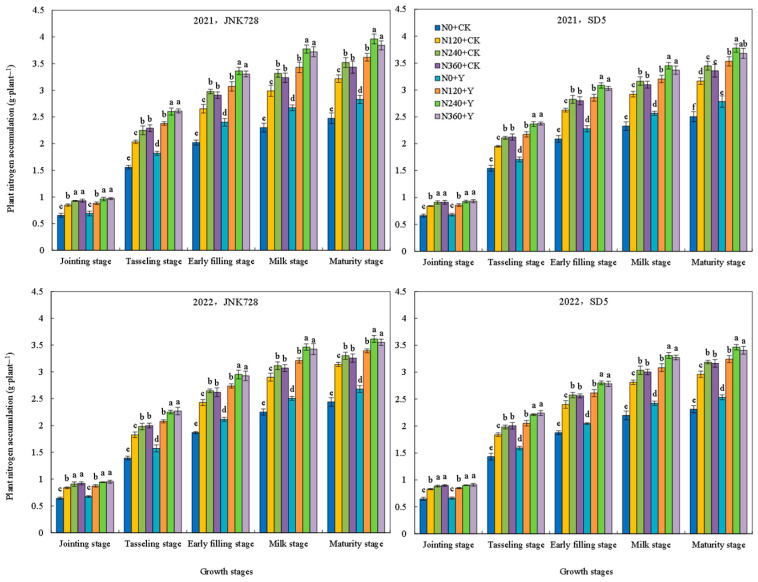
Effects of nitrogen fertilizer and chemical regulation on plant nitrogen accumulation in 2021 and 2022. Columns within the same growth stage marked with different letters indicate statistically significant differences at *p* < 0.05. Vertical bars mean ± SE. JNK728 and SD5 indicate maize varieties Jingnongke 728 and Saide 5, respectively. N0 + CK, N120 + CK, N240 + CK, and N360 + CK represent nitrogen application rates of 0, 120, 240, and 360 kg ha^−1^ combined with water application, while N0 + Y, N120 + Y, N240 + Y, and N360 + Y represent the nitrogen application rates of 0, 120, 240, and 360 kg ha^−1^ combined with plant growth regulator.

The nitrogen accumulation in plants consistently increased throughout the growth period (Figure 22). It rose rapidly from the jointing stage to the milk stage, then slowed down gradually, reaching the maximum at the maturity stage. Nitrogen application notably enhanced nitrogen accumulation at all growth stages, with no significant difference between N240 and N360. Chemical regulation significantly improved plant nitrogen accumulation in maize throughout the growth period over two years. These results indicated that nitrogen fertilizer and chemical regulation promoted plant nitrogen accumulation under high planting density, maintaining sufficient nitrogen supply during the growth period.

#### 2.3.2. Nitrogen Translocation

Nitrogen application significantly increased post-anthesis nitrogen translocation amount from vegetative organs in maize. Compared with N0, the nitrogen translocation amount of leaf and stem + sheath under N120, N240, and N360 increased by 7.9%, 15.3%, and 11.9%, and 6.6%, 14.3%, and 11.6%, respectively (Table 5). However, increasing nitrogen rate reduced the contribution rate of the leaf and stem + sheath nitrogen translocation amount to grain. Chemical regulation enhanced the post-anthesis nitrogen translocation amount from vegetative organs and its contribution rate to grain. Compared with CK, chemical regulation increased the nitrogen translocation amount of the leaf and stem + sheath by 15.1% and 14.7%, and their contribution rates to grain by 3.7% and 3.3%, respectively. These results indicated that nitrogen fertilizer and chemical regulation improved nitrogen translocation and promoted nitrogen allocation to grains under high planting density.

#### 2.3.3. Nitrogen Accumulation in Different Organs at Maturity Stage

Nitrogen application rate and chemical regulation significantly affected nitrogen accumulation in different organs at the maturity stage under high planting density (Table 6). Compared with N0, the nitrogen application of N120, N240, and N360 increased nitrogen accumulation in the leaf by 22.5%, 30.1%, and 26.5%, in the stem + sheath by 24.0%, 30.6%, and 27.8%, in the cob + ear bract by 30.6%, 41.0%, and 41.6%, and in grain by 29.1%, 40.5%, and 37.3%, respectively. Chemical regulation improved nitrogen accumulation in the leaf, the stem + sheath, the cob + ear bract, and grain by 9.2%, 9.0%, 7.4%, and 11.0% compared with CK. These results indicated that nitrogen fertilizer and chemical regulation promoted nitrogen accumulation and distribution in the vegetative organs and grains of high-density at the maturity stage.

#### 2.3.4. Nitrogen Utilization

Nitrogen application and chemical regulation significantly affected maize nitrogen use efficiency (NUE) under high planting density (Table 7). With increasing nitrogen application rate, nitrogen uptake efficiency, nitrogen partial factor productivity, nitrogen agronomic efficiency and nitrogen use efficiency decreased. Chemical regulation increased nitrogen uptake efficiency, nitrogen partial factor productivity, nitrogen agronomic efficiency and nitrogen use efficiency by 3.1%, 5.9%, 5.6%, and 6.6%, respectively, compared with CK. These results indicated that nitrogen application reduced nitrogen utilization, while chemical regulation improved maize nitrogen use efficiency under high planting density.

#### 2.3.5. Nitrogen Metabolism Enzymes Activities in Grain

**Figure 23:**
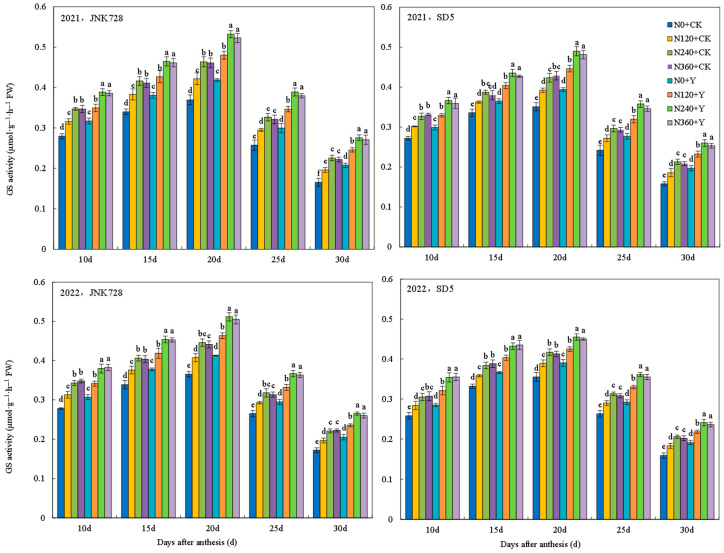
Effects of nitrogen fertilizer and chemical regulation on the glutamate synthetase (GS) activity in grain in 2021 and 2022. Columns within the same days after anthesis marked with different letters indicate statistically significant differences at *p* < 0.05. Vertical bars mean ± SE. JNK728 and SD5 indicate maize varieties Jingnongke 728 and Saide 5, respectively. N0 + CK, N120 + CK, N240 + CK, and N360 + CK represent nitrogen application rates of 0, 120, 240, and 360 kg ha^−1^ combined with water application, while N0 + Y, N120 + Y, N240 + Y, and N360 + Y represent the nitrogen application rates of 0, 120, 240, and 360 kg ha^−1^ combined with plant growth regulator.

**Figure 24:**
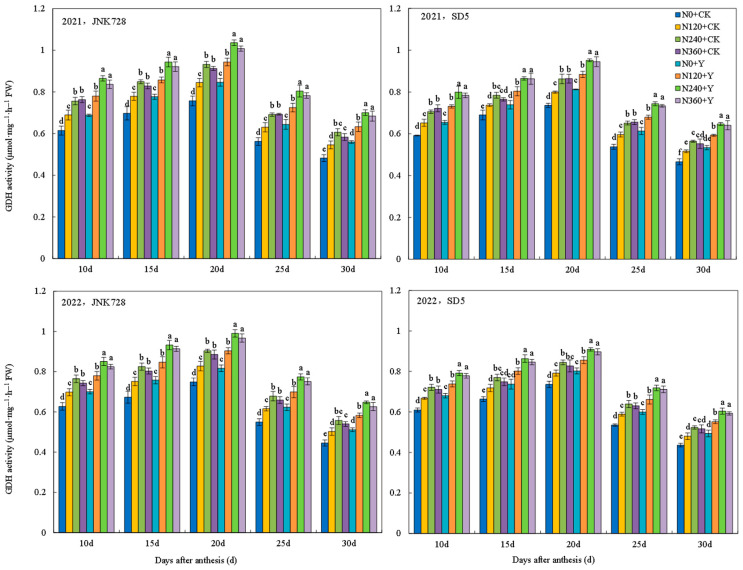
Effects of nitrogen fertilizer and chemical regulation on the glutamate dehydrogenase (GDH) activity in grain in 2021 and 2022. Columns within the same days after anthesis marked with different letters indicate statistically significant differences at *p* < 0.05. Vertical bars mean ± SE. JNK728 and SD5 indicate maize varieties Jingnongke 728 and Saide 5, respectively. N0 + CK, N120 + CK, N240 + CK, and N360 + CK represent nitrogen application rates of 0, 120, 240, and 360 kg ha^−1^ combined with water application, while N0 + Y, N120 + Y, N240 + Y, and N360 + Y represent the nitrogen application rates of 0, 120, 240, and 360 kg ha^−1^ combined with plant growth regulator.

**Figure 25:**
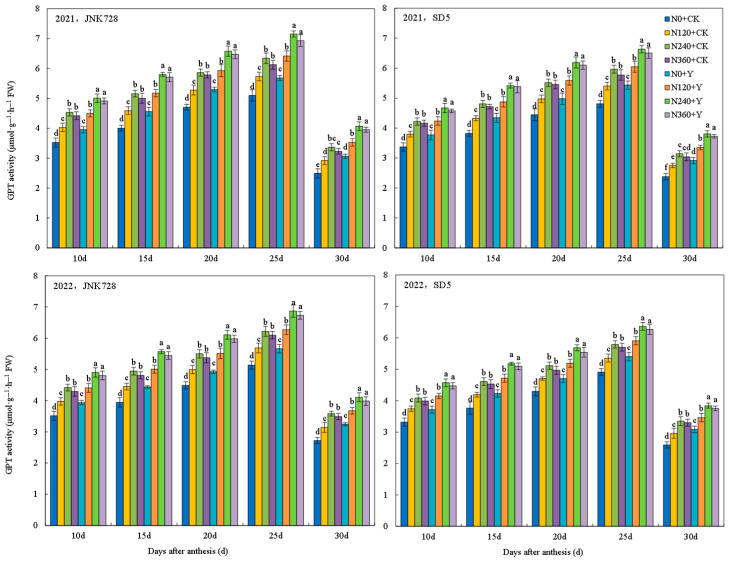
Effects of nitrogen fertilizer and chemical regulation on the glutamate-pyruvate transaminase (GPT) activity in grain in 2021 and 2022. Columns within the same days after anthesis marked with different letters indicate statistically significant differences at *p* < 0.05. Vertical bars mean ± SE. JNK728 and SD5 indicate maize varieties Jingnongke 728 and Saide 5, respectively. N0 + CK, N120 + CK, N240 + CK, and N360 + CK represent nitrogen application rates of 0, 120, 240, and 360 kg ha^−1^ combined with water application, while N0 + Y, N120 + Y, N240 + Y, and N360 + Y represent the nitrogen application rates of 0, 120, 240, and 360 kg ha^−1^ combined with plant growth regulator.

Glutamate synthetase (GS), glutamate dehydrogenase (GDH), and glutamate-pyruvate transaminase (GPT) are key enzymes regulating maize nitrogen metabolism. The activities of GS, GDH, and GPT in grains under different treatments all followed a single-peak curve during the filling stage, for which GS and GDH peaked at 20 days after anthesis (DAA), while GPT peaked at 25 DAA (Figure 23, Figure 24 and Figure 25). Nitrogen fertilizer and chemical regulation significantly affected nitrogen metabolism enzymes activities under high planting density. With increasing nitrogen rate, the activities of GS, GDH, and GPT significantly increased during grain filling stages, but no further increase was observed under excessive nitrogen (N360). Chemical regulation increased the activities of GS (20 DAA), GDH (20 DAA), and GPT (25 DAA) by 12.7%, 9.8%, and 11.3%, respectively. These results indicated that appropriate nitrogen application and chemical regulation can significantly improve the activities of grain GS, GDH, and GPT under high planting density, enhancing grain nitrogen metabolism capacity.

### 2.4. Yield

**Figure 26:**
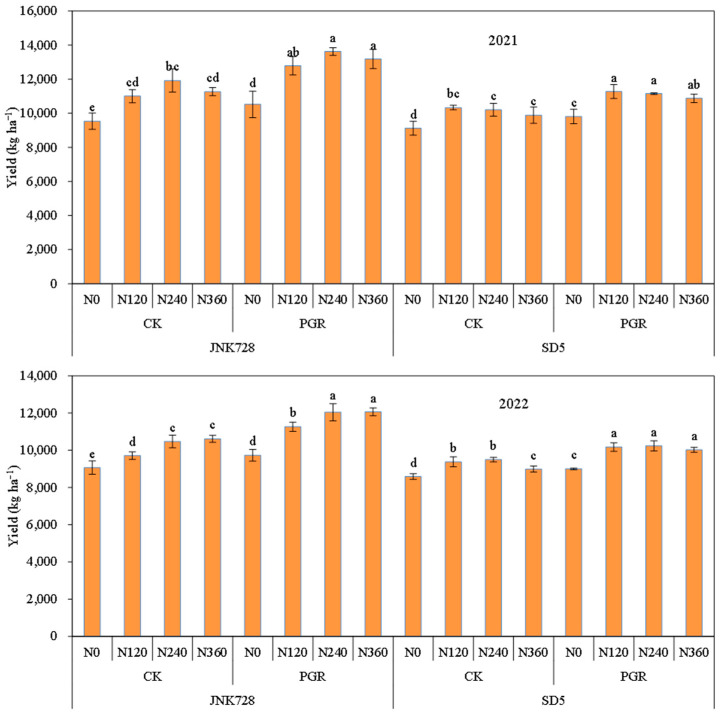
Effects of nitrogen fertilizer and chemical regulation on yield in 2021 and 2022. Columns within the same variety marked with different letters indicate statistically significant differences at *p* < 0.05. Vertical bars mean ± SE. JNK728 and SD5 indicate maize varieties Jingnongke 728 and Saide 5, respectively. N0, N120, N240, and N360 represent nitrogen application rates of 0, 120, 240, and 360 kg ha^−1^, respectively. PGR and CK represent the application of plant growth regulator and water, respectively.

Nitrogen application and chemical regulation significantly affected maize yield under high planting density (Figure 26). Nitrogen application notably increased yield: compared with N0, yields under N120, N240, and N360 increased by 16.4%, 20.3%, and 16.0% in 2021 and 11.4%, 16.1%, and 14.6% in 2022, with N240 giving the highest yield. Chemical regulation raised yield by 12.0% in 2021 and 10.8% in 2022 compared with CK. JNK728 had significantly higher yield than SD5. For yield components, nitrogen application increased grain number per ear and 1000-grain weight but barely affected ear number per unit area. Chemical regulation also improved grain number per ear (8.9% in 2021 and 10.3% in 2022) and 1000-grain weight (5.0% in 2021 and 4.1% in 2022).

## 3. Discussion

### 3.1. Effects of Nitrogen Fertilizer and Chemical Regulation on Photosynthetic Characteristics and Canopy Structure Under High Planting Density

Photosynthesis serves as the fundamental basis for plant growth and yield formation. High planting density typically leads to a decline in leaf chlorophyll content and photosynthetic performance [22]. In this study, nitrogen fertilizer significantly enhanced key photosynthetic parameters, including SPAD values, Pn, Fv/Fm, and the activities of RuBPcase and PEPcase. However, the excessive nitrogen application did not confer additional benefits and even showed a declining trend. Meanwhile, chemical regulation increased these photosynthetic traits, likely by modulating plant growth and delaying leaf senescence [23]. This suggests that appropriate nitrogen and chemical regulation effectively alleviates photosynthetic suppression under high planting density.

The canopy structure is a critical determinant of light distribution and overall photosynthetic capacity [24]. An optimal canopy architecture improves light interception and ventilation, thereby enhancing photosynthetic efficiency and yield [25]. Our study revealed that nitrogen application increased the Leaf Area Index (LAI) and light interception of the canopy. However, excessive nitrogen led to an overly dense canopy, significantly reducing light transmittance that likely accelerated lower leaf senescence [26]. Chemical regulation could induce a more compact plant type [20]. In this study, chemical regulation reduced LAI from the jointing stage to early filling stages, thereby improving light transmittance to the middle and lower canopy layers. Subsequently, maintaining a higher LAI from the early filling to maturity stages may caused by delaying leaf senescence [27]. Therefore, chemical regulation increases the photosynthetic capacity of maize populations by optimizing canopy structure, thereby establishing a foundation for dry matter accumulation and yield.

### 3.2. Effects of Nitrogen Fertilizer and Chemical Regulation on Lodging Resistance Characteristics Under High Planting Density

Lodging remains a critical constraint to realizing the maize yield potential under high planting density [12]. As crucial indicators of stem mechanical strength, bending resistance and rind penetration strength are closely linked to lodging resistance in maize [12]. In this study, nitrogen application at the N120 and N240 levels significantly enhanced bending strength and rind penetration strength, demonstrating that nitrogen fertilizer promotes stem growth, improves mechanical strength, and consequently enhances the lodging resistance of maize under high planting density [28]. However, excessive nitrogen application reduces mechanical strength, which may be due to the inadequate utilization of carbohydrates for stem formation, resulting in a weak stem structure [29]. In this study, chemical regulation significantly enhanced the bending strength and rind penetration strength of maize stem. This improvement is likely attributable to the plant growth regulators modulating plant metabolism, promoting the accumulation of structural components, and improving stem organization, thereby reinforcing the mechanical strength [30].

Plant height, ear height, and center of gravity height are key morphological traits influencing lodging resistance in maize, and are negatively correlated with lodging resistance [28]. Lodging frequently occurs at the basal internodes in maize; thus, their morphological traits play a critical role for lodging resistance. Generally, shorter and thicker basal internodes are associated with superior lodging resistance [31]. In this study, increasing nitrogen application rates increased plant height, ear height, and the center of gravity height, along with the length and diameter of the third basal internode. However, the increases in plant height and ear height were not statistically significant. This is primarily attributed to the high planting density of this experiment accelerating stem elongation, which reduced the accumulation of cellulose and lignin and consequently led to poorer stem plumpness [32]. Under these conditions, the applied nitrogen was mainly attributed to increasing the carbohydrate content in the stem to enhance its strength, rather than to promote stem elongation. In this study, chemical regulation significantly reduced the length of basal internodes, plant height, ear height, and center of gravity height, while increasing stem diameter. This indicates that the plant growth regulator suppressed stem elongation and promoted the radial growth of basal internodes, resulting in a shorter, sturdier plant figure that enhances lodging resistance, consistent with findings by Ahmad et al. [19]. The improved plant and basal internode morphology effectively reduced the lodging risk in maize, which is fundamental to achieving high yield.

The dry weight per unit length of internodes reflects the extent of stem plumpness [31]. In this study, appropriate nitrogen fertilization combined with chemical regulation increased the dry weight per unit length of the internode. This improvement is likely attributed to enhanced photoassimilate accumulation in the stem, thereby improving stem plumpness [33]. A higher dry weight per unit length indicates greater dry matter accumulation, which consequently contributes to increased lodging resistance [31]. Therefore, the mechanical strength of the stem is underpinned by its biochemical composition. Cellulose, hemicellulose, and lignin are the primary structural components of cell walls and play a critical role in maintaining stem mechanical strength [34]. Water-soluble carbohydrates in the internodes not only serve as a crucial reserve substance during the grain filling stage but also contribute to the stem mechanical strength [33,35]. In this study, the combined application of N120 or N240 with chemical regulation increased the contents of soluble sugars, cellulose, hemicellulose, and lignin in the third basal internode of maize, thereby enhancing stem mechanical strength and improving lodging resistance. This indicates that the photosynthetic capacity of lower canopy leaves influences carbohydrate accumulation in the stem, thereby affecting stem mechanical strength [36]. Therefore, in this study, nitrogen and chemical regulation presumably improved carbohydrate content in the internodes by optimizing canopy structure and enhancing the photosynthetic performance of leaves at and below the ear layer. However, excessive nitrogen application led to a reduction in carbohydrate content in the stem. This may be due to the stem not fully utilizing photosynthates produced by leaves, thereby inhibiting the accumulation of carbohydrates in the stem [29]. As a key substance maintaining stem mechanical strength, lignin’s biosynthesis is closely associated with the activities of PAL, TAL, CAD, and 4CL [37]. In this study, the combined application of nitrogen rates (N120 and N240) with chemical regulation increased activities of these enzymes, which promotes lignin biosynthesis. Consequently, the combination of N120/N240 and chemical regulation promotes structural component accumulation in stems, thereby enhancing the stem lodging resistance.

In terms of the stem anatomical structure, stem strength largely depends on the mechanical tissue of the adjacent peripheral vascular bundles [32]. Studies have demonstrated that the vascular bundle system fulfills the functions of long-distance transport and mechanical support, playing a crucial role in the lodging resistance of maize [30]. Under high planting density, the stem rind thickness, vascular bundle sheath’s thickness, and vascular bundle number are significantly reduced, thereby decreasing the lodging resistance of maize [12]. In this study, nitrogen application (N120 and N240) and chemical regulation significantly increased stem cortex thickness, mechanical tissue thickness, and mechanical cell layer number, and increased the number and area of large and small vascular bundles, thereby enhancing stem strength and the lodging resistance of maize. Furthermore, as the conducting tissue, the structure of the vascular bundle plays a vital role in the transport of photosynthates in crops [38]. Therefore, the increase in the number and area of vascular bundles induced by nitrogen fertilizer and chemical regulation in this study not only enhances maize lodging resistance but also helps maintain high nutrient transport capacity, thereby promoting grain filling and yield improvement.

### 3.3. Effects of Nitrogen Fertilizer and Chemical Regulation on Nitrogen Uptake, Utilization, and Yield Formation Under High Planting Density

Nitrogen, as a crucial nutrient element during maize growth, plays a vital role in ear growth and yield formation [39]. In this study, the maximum nitrogen accumulation in plants was achieved under the treatment of nitrogen application rate N240 combined with chemical regulation. This may be due to the improvement of root growth and root activity by nitrogen fertilizer and chemical regulation, which promotes the root absorption of soil nitrogen, thereby facilitating the yield increase [17,40,41]. The process of grain formation in maize is related to nitrogen accumulation in grains, which ultimately determines grain yield [42]. Studies have shown that grain nitrogen is supplied by both the remobilization of pre-anthesis reserves in vegetative organs and direct post-anthesis nitrogen assimilation [43]. During grain filling, nitrogen remobilization serves as a major source for grain nitrogen accumulation. Studies have indicated that leaf nitrogen is also vital for photosynthesis, and excessive remobilization leads to leaf senescence and reduces photosynthetic output [44]. Optimizing the balance between nitrogen translocation and photosynthetic retention is therefore crucial for a high yield in maize. In this study, the nitrogen rate at N240 combined with chemical regulation promoted nitrogen absorption, distribution, and translocation, while also increasing nitrogen accumulation in leaves, thereby maintaining high photosynthetic activity and ultimately enhancing grain yield. Grain nitrogen is mostly derived from post-anthesis nitrogen absorption. Enhancing post-anthesis nitrogen absorption can sustain photosynthetic capacity during grain filling, and thereby contributes to increased yield [45]. Consequently, modulating nitrogen uptake and remobilization improves nitrogen utilization in maize. Furthermore, in this study, nitrogen fertilizer and chemical regulation increased the activities of GS, GDH, and GPT in grains, and enhanced grain nitrogen metabolism, further promoting nitrogen utilization efficiency and providing a foundation for increased maize yield. In this study, chemical regulation significantly increased the grain number per ear, 1000-grain weight, and yield in maize under high planting density. Nitrogen application remarkably improved maize yield and its components, while no significant difference was observed between nitrogen rates N240 and N360. Consistent with previous findings, no further yield improvement was observed with excessive nitrogen fertilizer application in maize [46]. In this study, the combination of nitrogen fertilizer and chemical regulation simultaneously meets the photosynthetic demand of maize and nitrogen utilization, which is of great significance for improving maize yield and nitrogen use efficiency.

## 4. Materials and Methods

### 4.1. Experimental Site

**Figure 27:**
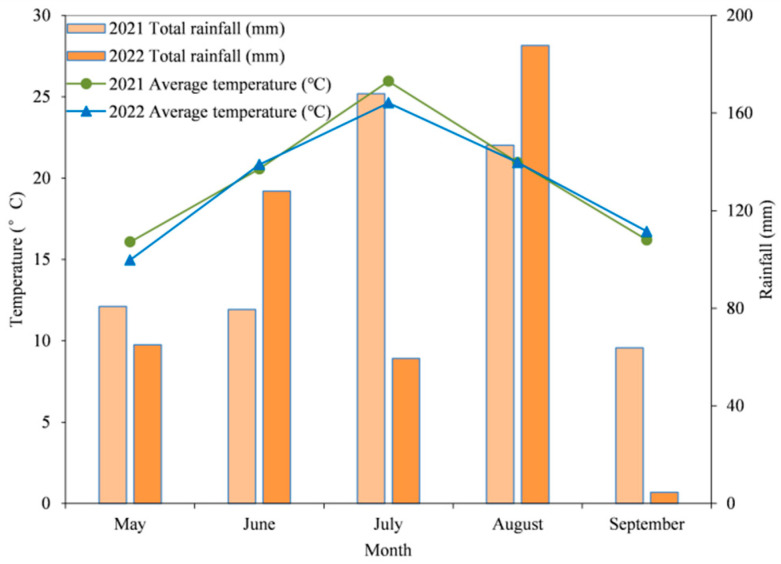
Meteorological conditions during the maize growing seasons.

The field experiment was conducted in Daowai District of Harbin city (45°75′ N, 126°63′ E), Heilongjiang Province, in 2021 and 2022. The experimental soil was characterized by a typical chernozem soil and has been managed under a continuous maize system. The properties of the topsoil layer (0–20 cm, pH 6.75) were determined as follows: organic matter, available N, available phosphorus P, and available potassium K were 1.773 g kg^−1^, 220.38 mg kg^−1^, 59.85 mg kg^−1^, and 131.24 mg kg^−1^, respectively. This region experienced a temperate continental monsoon climate. The climatic parameters include 4.3 °C of mean annual temperature, 569.1 mm of mean annual precipitation, 2642.1 h of sunshine, 1324.3 mm of evaporation, and 140–150 days of frost-free period. Figure 27 shows the meteorological conditions for maize growing seasons of 2021 and 2022.

### 4.2. Experimental Design and Field Management

A split-split plot arrangement was employed, incorporating three factors: maize cultivar (main plot), chemical regulation (subplot), and nitrogen (N) application rate (sub-subplot). The main plots consisted of two cultivars: Jingnongke 728 (JNK728) and Saide 5 (SD5). Subplots received one of two chemical treatments: a foliar application of a plant growth regulator (PGR, 450 L ha^−1^ of 30% diethyl aminoethyl hexanoate · ethephon) at the seven-leaf stage, or a control (CK) with water only. The sub-subplots applied nitrogen at rates of 0, 120, 240, and 360 kg ha^−1^. This structure resulted in 16 treatment combinations, each replicated three times. A planting density of 90,000 plants ha^−1^ was established through manual sowing (with a depth of 5 cm) in late April. The plot dimensions were 10 rows, 8 m in length, with 65 cm row spacing. Nitrogen was supplied equally between a basal application at sowing and a topdressing at jointing. Phosphorus (P_2_O_5_) and potassium (K_2_O) were fully applied as basal dressing at 100 kg ha^−1^ each. The crop relied on rainfall, with no irrigation applied. Field management, including pest, weed, and disease control, followed standard local practices. Harvesting occurred on September 25 each year.

### 4.3. Sampling and Measurements

#### 4.3.1. Photosynthesis and Canopy Parameters

Leaf chlorophyll content, expressed as SPAD values, was determined with a SPAD-502 m (Minolta, Tokyo, Japan). At the jointing, tasseling, early filling, milking, and maturity stages, three uniformly growing plants per treatment were selected. Functional leaves (the third leaf below the apex before tasseling and the ear leaf after tasseling) were used for measurement. Leaf net photosynthetic rate (Pn) was measured between 9:00 and 11:00 am on clear days, using a Li-6400 portable photosynthesis system (LI-COR, Lincoln, NE, USA). The same leaf selection procedure as for SPAD determination was followed. Using the same leaf sampling method, the maximum quantum yield (Fv/Fm) was determined according to the formula: Fv/Fm = (Fm − F_0_)/Fm. The initial fluorescence (F_0_) and maximum fluorescence (Fm) were measured after the leaves were completely dark-adapted post 21:00. For the same set of leaves, activities of RuBPCase and PEPCase were determined according to the methods described by Ding et al. [47].

At five key growth stages (jointing, tasseling, early filling, milking, and maturity), three representative plants per treatment were sampled. The leaf area of individual plants was estimated as length × width × factor (coefficients of 0.75 and 0.5 for unfolded and folded leaves, respectively). LAI was defined as the ratio of the total green leaf area to the corresponding ground surface area. During the early filling stage, a CI-110 plant canopy analyzer was employed to measure light transmittance at three vertical positions: the upper canopy, ear layer, and lower canopy (30 cm above ground). Three replicates were performed per plot.

#### 4.3.2. Lodging Resistance Characteristics

At the milking stage, three maize plants with uniform growth were sampled from each treatment. Plant height, ear height, and center of gravity height were measured using a measuring rod. These parameters were defined as the vertical distance from the ground level to the tassel’s highest point, the node bearing the ear, and the plant’s balance point, respectively. The ear height coefficient was computed as the percentage of ear height relative to plant height.

During the early filling stage, three uniformly growing plants per treatment were sampled. The third basal internode’s length and diameter were determined using a ruler and a vernier caliper. Subsequently, the samples were oven-dried at 105 °C for 30 min, followed by drying at 80 °C to constant weight. The dry weight per unit length (g cm^−1^) of the internode was determined as the ratio of dry weight to length.

The bending strength and rind penetration strength of the third basal internode were evaluated using an AWOS-SL04 stem strength tester (AWOS Technology, Shijiazhuang, China). A bending probe and a penetration probe were applied perpendicularly to the midpoint of the internode at a constant speed until fracture or piercing occurred. The maximum force recorded at failure was used to determine mechanical strength.

The lodging resistance index was calculated as the bending strength of the third internode divided by the height of the stem’s center of gravity [48]. At physiological maturity, the lodged plants in each plot were counted. The lodging rate was calculated as the percentage of lodged plants relative to the total plant count per plot.

The soluble sugar content in the third basal internode was quantified by the anthrone colorimetric method [49]. Cellulose, hemicellulose, and lignin, were analyzed according to the Van Soest detergent method [50]. Enzyme activities of PAL, TAL, CAD, and 4CL were assayed following procedures described by Liu et al. [37].

At the early filling stage, segments about 1.5 cm in length were excised from the middle of the third basal internode and fixed in Carnoy’s solution for 24 h. The samples were then stored in 70% ethanol. Hand-cut sections were prepared, stained with safranin, and observed under a fluorescence microscope. Images were captured, and parameters including cortex thickness, mechanical thickness, mechanical cell layer number, and the number and area of large and small vascular bundles were measured using the built-in image analysis system [12].

#### 4.3.3. Nitrogen Utilization and Grain Yield

At five key growth stages, three representative plants per treatment were sampled. These plants were separated into different organs (leaf, stem + leaf sheath, cob + bract, and grain). All samples were first oven-dried at 105 °C for 30 min and then dried at 80 °C until a constant weight. The dried samples were ground into a fine powder, and the nitrogen concentration in the whole plant and individual organs was determined using the semi-micro Kjeldahl method. A series of nitrogen-related parameters were calculated according to established methodologies [51,52,53]:

Plant nitrogen accumulation (g plant^−1^) = Plant nitrogen concentration (%) × Plant dry weight (g plant^−1^).

Organ nitrogen accumulation (g plant^−1^) = Organ nitrogen concentration (%) × Organ dry weight (g plant^−1^).

Nitrogen translocation amount (g plant^−1^) = Nitrogen accumulation in vegetative organs at anthesis (g plant^−1^-Nitrogen accumulation in vegetative organs at maturity (g plant^−1^).

Contribution of nitrogen translocation amount to grain (%) = [Nitrogen translocation amount (g plant^−1^)/Nitrogen accumulation in grains at maturity (g plant^−1^)] × 100.

Nitrogen uptake efficiency (kg kg^−1^) = Plant nitrogen accumulation (kg ha^−1^)/Nitrogen application rate (kg ha^−1^).

Nitrogen partial factor productivity (kg kg^−1^) = Grain yield (kg ha^−1^)/Nitrogen application rate (kg ha^−1^).

Nitrogen agronomic efficiency (kg kg^−1^) = [Grain yield under nitrogen fertilization (kg ha^−1^)-Grain yield without nitrogen fertilization (kg ha^−1^)]/Nitrogen application rate (kg ha^−1^).

Nitrogen use efficiency (%) = [Nitrogen accumulation under nitrogen fertilization (kg ha^−1^)-Nitrogen accumulation without nitrogen fertilization (kg ha^−1^)]/Nitrogen application rate (kg ha^−1^) × 100.

At 10, 20, 30, 40, and 50 days after anthesis, three ears were collected from each treatment. Grains from the middle section of the ears were excised, immediately frozen in liquid nitrogen, and stored at −80 °C for subsequent enzyme analysis. Glutamate synthetase (GS), glutamate dehydrogenase (GDH), and glutamate-pyruvate transaminase (GPT) activities were measured according to Wang et al. [54].

Grain yield and yield components were evaluated by harvesting the central three rows at maturity. The grain yield was corrected to 14% moisture content, while grain number per ear and 1000-grain weight were recorded.

### 4.4. Statistical Analysis

To evaluate the treatment effects of nitrogen fertilizer and chemical regulation, the collected data were subjected to statistical examination. This process involved two-way analysis of variance (ANOVA) and a least significant difference (LSD) test at the *p* < 0.05 level, assessing key parameters including canopy photosynthesis, lodging resistance, nitrogen use efficiency, and grain yield. All analyses were performed with SPSS Statistics 21.0 (SPSS Inc., Chicago, IL, USA). The figures presented in this study were prepared with Microsoft Excel 2010.

## 5. Conclusions

Nitrogen fertilizer (240 kg N ha^−1^) combined with chemical regulation (30% diethyl aminoethyl hexanoate · ethephon) improved leaf photosynthetic capacity and canopy structure, and enhanced stem lodging resistance, thereby ultimately increasing maize yield and nitrogen use efficiency. This cultivation measure effectively coordinated the conflict between high yield and lodging risk, providing a scientific basis for high-yield cultivation under dense planting conditions.

## Figures and Tables

**Table 1:** Effect of nitrogen fertilizer and chemical regulation on plant morphology.

	2021				2022			
Treatment	Plant Height (cm)	Ear Height (cm)	Ear Height Coefficient (%)	Center of Gravity Height (cm)	Plant Height (cm)	Ear Height (cm)	Ear Height Coefficient (%)	Center of Gravity Height (cm)
Nitrogen application								
N0	278.1 b	111.5 c	40.08 b	105.6 c	268.4 c	106.0 c	39.47 b	102.6 d
N120	279.9 b	115.3 bc	41.14 ab	108.9 bc	272.8 c	109.2 c	39.99 b	106.0 c
N240	282.5 b	118.3 ab	41.81 a	111.7 b	277.8 b	118.0 b	41.35 a	109.3 b
N360	289.6 a	122.0 a	42.09 a	117.6 a	287.8 a	120.9 a	41.99 a	115.6 a
Chemical regulation								
CK	290.4 a	122.9 a	42.28 a	116.9 a	282.8 a	118.1 a	41.74 a	113.0 a
PGR	274.6 b	110.6 b	40.29 b	104.7 b	270.7 b	107.4 b	39.66 b	103.8 b
Variety								
JNK728	289.3 a	123.0 a	42.48 a	113.1 a	284.8 a	117.0 a	41.05 a	111.3 a
SD5	275.8 b	110.5 b	40.08 b	108.4 b	268.7 b	108.5 b	40.35 a	105.5 b
Sources of variation								
V	**	**	**	**	**	**	NS	**
N	*	**	NS	**	**	**	*	**
C	**	**	*	**	**	**	**	**
V × N	NS	NS	NS	NS	NS	NS	NS	NS
V × C	*	**	NS	NS	**	*	NS	NS
N × C	NS	NS	NS	NS	NS	NS	NS	NS
V × N × C	NS	NS	NS	NS	NS	NS	NS	NS

N0, N120, N240, and N360 indicate nitrogen application rates at 0, 120, 240, and 360 kg ha^−1^, respectively. PGR and CK indicate spraying plant growth regulator and water, respectively. JNK728 and SD5 indicate maize varieties Jingnongke 728 and Saide 5, respectively. V, N, and C indicate variety, nitrogen application, and chemical regulation, respectively. Different letters in the same column indicate a significant difference between different treatments (*p* < 0.05). * and ** indicate significant differences at the 0.05 and 0.01 probability levels, respectively, and NS indicates no significant difference.

**Table 2:** Effect of nitrogen fertilizer and chemical regulation on the third basal internode morphology.

		2021			2022	
Treatment	Internode Length (cm)	Stem Diameter (mm)	Dry Weight per Unit Length (g·cm^−1^)	Internode Length (cm)	Stem Diameter (mm)	Dry Weight per Unit Length (g·cm^−1^)
Nitrogen application						
N0	19.19 c	23.35 c	0.362 c	18.71 c	21.79 c	0.343 c
N120	19.77 bc	25.13 b	0.418 a	19.17 bc	23.25 b	0.386 a
N240	20.37 b	26.86 a	0.403 ab	19.67 b	24.96 a	0.382 a
N360	21.77 a	26.44 a	0.387 b	21.03 a	24.91 a	0.361 b
Chemical regulation						
CK	21.31 a	24.62 b	0.376 b	20.65 a	22.93 b	0.354 b
PGR	19.24 b	26.27 a	0.409 a	18.64 b	24.53 a	0.382 a
Variety						
JNK728	20.92 a	25.21 a	0.381 b	20.17 a	23.49 a	0.357 b
SD5	19.63 b	25.68 a	0.404 a	19.12 b	23.97 a	0.379 a
Sources of variation						
V	*	NS	**	*	NS	**
N	**	**	**	**	**	**
C	**	**	**	**	**	**
V × N	NS	NS	NS	NS	NS	NS
V × C	*	NS	NS	NS	NS	NS
N × C	NS	NS	NS	NS	NS	NS
V × N × C	NS	NS	NS	NS	NS	NS

N0, N120, N240, and N360 indicate nitrogen application rates at 0, 120, 240, and 360 kg ha^−1^, respectively. PGR and CK indicate spraying plant growth regulator and water, respectively. JNK728 and SD5 indicate maize varieties Jingnongke 728 and Saide 5, respectively. V, N, and C indicate variety, nitrogen application, and chemical regulation, respectively. Different letters in the same column indicate a significant difference between different treatments (*p* < 0.05). * and ** indicate significant differences at the 0.05 and 0.01 probability levels, respectively, and NS indicates no significant difference.

**Table 3:** Correlation analysis of stem mechanical characteristics with internode carbohydrate contents.

Index	Soluble Sugar Content	Cellulose Content	Hemicellulose Content	Lignin Content
Bending strength	0.885 **	0.969 **	0.851 **	0.957 **
Rind penetration strength	0.934 **	0.983 **	0.902 **	0.945 **
Lodging resistance index	0.980 **	0.902 **	0.965 **	0.795 **
Lodging rate	−0.925 **	−0.972 **	−0.891 **	−0.938 **

** indicate significant differences at the 0.01 probability levels.

**Table 4:** Effect of nitrogen fertilizer and chemical regulation on the anatomical structure of the stem in 2021.

Treatment	Cortex Thickness (μm)	Mechanical Thickness (μm)	Mechanical Cell Layers	Big Vascular Bundle Number	Small Vascular Bundle Number	Big Vascular Bundle Area (mm^2^)	Small Vascular Bundle Area (mm^2^)
Nitrogen application							
N0	48.93 c	16.98 c	1.37 c	235.33 c	331.49 c	20.19 c	7.65 c
N120	59.19 a	21.07 a	1.72 a	257.42 a	392.46 a	23.07 a	9.24 a
N240	57.72 a	20.56 a	1.68 a	259.45 a	383.99 a	22.71 a	9.55 a
N360	54.15 b	19.06 b	1.55 b	249.37 b	355.85 b	21.42 b	8.74 b
Chemical regulation							
CK	49.44 b	17.88 b	1.45 b	240.87 b	339.04 b	20.45 b	8.10 b
PGR	61.12 a	20.96 a	1.71 a	260.42 a	396.36 a	23.25 a	9.49 a
Variety							
JNK728	53.08 b	16.67 b	1.37 b	243.65 b	361.82 a	22.46 a	8.43 b
SD5	57.48 a	22.17 a	1.79 a	257.64 a	373.58 a	21.24 b	9.15 a
Sources of variation							
V	**	**	**	*	NS	*	**
N	**	**	**	**	**	**	**
C	**	**	**	**	**	**	**
V × N	NS	NS	NS	NS	NS	NS	NS
V × C	NS	NS	NS	NS	NS	NS	NS
N × C	NS	NS	NS	NS	NS	NS	NS
V × N × C	NS	NS	NS	NS	NS	NS	NS

N0, N120, N240, and N360 indicate nitrogen application rates at 0, 120, 240, and 360 kg ha^−1^, respectively. PGR and CK indicate spraying plant growth regulator and water, respectively. JNK728 and SD5 indicate maize varieties Jingnongke 728 and Saide 5, respectively. V, N, and C indicate variety, nitrogen application, and chemical regulation, respectively. Different letters in the same column indicate a significant difference between different treatments (*p* < 0.05). * and ** indicate significant differences at the 0.05 and 0.01 probability levels, respectively, and NS indicates no significant difference.

**Table 5:** Effect of nitrogen fertilizer and chemical regulation on the post-anthesis nitrogen translocation amount from vegetative organs and its contribution rate to grain.

	2021				2022			
Treatment	Nitrogen Translocation Amount (g·Plant^−1^)		Contribution of Nitrogen Translocation Amount to Grain (%)		Nitrogen Translocation Amount (g·Plant^−1^)		Contribution of Nitrogen Translocation Amount to Grain (%)	
	Leaf	Stem + Sheath	Leaf	Stem + Sheath	Leaf	Stem + Sheath	Leaf	Stem + Sheath
Nitrogen application								
N0	0.301 c	0.556 c	22.75 a	42.02 a	0.291 c	0.541 c	22.49 a	41.80 a
N120	0.328 b	0.595 b	19.18 b	34.78 b	0.311 b	0.575 b	18.63 b	34.43 b
N240	0.355 a	0.650 a	18.92 b	34.61 b	0.328 a	0.605 a	18.18 b	33.56 b
N360	0.343 ab	0.625 a	18.92 b	34.45 b	0.320 ab	0.600 ab	17.96 b	33.66 b
Chemical regulation								
CK	0.307 b	0.564 b	19.24 a	35.36 a	0.292 b	0.542 b	18.83 a	34.92 a
PGR	0.357 a	0.649 a	20.19 a	36.71 a	0.333 a	0.619 a	19.32 a	35.90 a
Variety								
JNK728	0.351 a	0.643 a	20.35 a	37.28 a	0.330 a	0.615 a	19.60 a	36.52 a
SD5	0.313 b	0.570 b	19.11 b	34.80 b	0.295 b	0.546 b	18.55 b	34.29 b
Sources of variation								
V	**	**	**	**	**	**	*	**
N	**	**	*	*	**	*	*	*
C	**	**	*	NS	**	**	NS	NS
V × N	NS	NS	NS	NS	*	NS	NS	NS
V × C	NS	*	NS	NS	*	NS	NS	NS
N × C	NS	NS	NS	NS	NS	NS	NS	NS
V × N × C	NS	NS	NS	NS	NS	NS	NS	NS

N0, N120, N240, and N360 indicate nitrogen application rates at 0, 120, 240, and 360 kg ha^−1^, respectively. PGR and CK indicate spraying plant growth regulator and water, respectively. JNK728 and SD5 indicate maize varieties Jingnongke 728 and Saide 5, respectively. V, N, and C indicate variety, nitrogen application, and chemical regulation, respectively. Different letters in the same column indicate a significant difference between different treatments (*p* < 0.05). * and ** indicate significant differences at the 0.05 and 0.01 probability levels, respectively, and NS indicates no significant difference.

**Table 6:** Effect of nitrogen fertilizer and chemical regulation on nitrogen accumulation in different organs at the maturity stage (g·plant^−1^).

	2021				2022			
Treatment	Leaf	Stem + Sheath	Cob + Ear Bract	Grain	Leaf	Stem + Sheath	Cob + Ear Bract	Grain
Nitrogen application								
N0	0.523 c	0.501 c	0.296 c	1.324 c	0.490 c	0.472 b	0.234 c	1.295 c
N120	0.633 b	0.613 b	0.392 b	1.711 b	0.607 b	0.593 a	0.302 b	1.671 b
N240	0.673 a	0.650 a	0.424 a	1.878 a	0.643 a	0.620 a	0.325 a	1.803 a
N360	0.650 ab	0.630 ab	0.436 a	1.814 a	0.630 ab	0.613 a	0.319 a	1.782 a
Chemical regulation								
CK	0.586 b	0.570 b	0.370 b	1.595 b	0.573 b	0.552 b	0.287 b	1.552 b
PGR	0.654 a	0.627 a	0.404 a	1.768 a	0.612 a	0.597 a	0.303 a	1.724 a
Variety								
JNK728	0.636 a	0.613 a	0.398 a	1.725 a	0.606 a	0.591 a	0.303 a	1.684 a
SD5	0.604 b	0.584 b	0.376 b	1.638 b	0.579 a	0.558 b	0.287 b	1.592 b
Sources of variation								
V	**	*	**	**	*	**	**	**
N	**	**	**	**	**	**	**	**
C	**	**	**	**	**	**	**	**
V × N	NS	NS	NS	NS	*	NS	NS	NS
V × C	*	NS	*	NS	NS	NS	NS	NS
N × C	NS	NS	NS	NS	NS	NS	NS	NS
V × N × C	NS	NS	NS	NS	NS	NS	NS	NS

N0, N120, N240, and N360 indicate nitrogen application rates at 0, 120, 240, and 360 kg ha^−1^, respectively. PGR and CK indicate spraying plant growth regulator and water, respectively. JNK728 and SD5 indicate maize varieties Jingnongke 728 and Saide 5, respectively. V, N, and C indicate variety, nitrogen application, and chemical regulation, respectively. Different letters in the same column indicate a significant difference between different treatments (*p* < 0.05). * and ** indicate significant differences at the 0.05 and 0.01 probability levels, respectively, and NS indicates no significant difference.

**Table 7:** Effect of nitrogen fertilizer and chemical regulation on nitrogen utilization.

	2021				2022			
Treatment	Nitrogen Uptake Efficiency (kg·kg^−1^)	Nitrogen Partial Factor Productivity (kg·kg^−1^)	Nitrogen Agronomic Efficiency (kg·kg^−1^)	Nitrogen Use Efficiency (%)	Nitrogen Uptake Efficiency (kg·kg^−1^)	Nitrogen Partial Factor Productivity (kg·kg^−1^)	Nitrogen Agronomic Efficiency (kg·kg^−1^)	Nitrogen Use Efficiency (%)
Nitrogen application								
N0								
N120	2.54 a	94.58 a	13.35 a	55.14 a	2.38 a	84.41 a	8.63 a	51.91 a
N240	1.38 b	48.85 b	8.23 b	38.59 b	1.27 b	44.00 b	6.11 b	33.95 b
N360	0.89 c	31.40 c	4.33 c	23.25 c	0.84 c	28.95 c	3.69 c	21.47 c
Chemical regulation								
CK	1.57 a	56.51 b	8.42 b	37.70 b	1.48 a	51.06 b	5.97 b	34.67 b
PGR	1.63 a	60.05 a	8.85 a	40.28 a	1.51 a	53.85 a	6.32 a	36.88 a
Variety								
JNK728	1.66 a	59.83 a	8.91 a	40.19 a	1.54 a	54.03 a	6.36 a	36.79 a
SD5	1.55 b	56.72 b	8.36 b	37.79 b	1.46 b	50.87 b	5.93 b	34.76 b
Sources of variation								
V	**	*	**	**	*	**	**	*
N	**	**	**	**	**	**	**	**
C	NS	**	*	**	NS	*	**	**
V × N	NS	*	NS	NS	NS	NS	*	NS
V × C	NS	NS	*	*	NS	NS	NS	*
N × C	NS	NS	NS	NS	NS	NS	NS	NS
V × N × C	NS	NS	NS	NS	NS	NS	NS	NS

N0, N120, N240, and N360 indicate nitrogen application rates at 0, 120, 240, and 360 kg ha^−1^, respectively. PGR and CK indicate spraying plant growth regulator and water, respectively. JNK728 and SD5 indicate maize varieties Jingnongke 728 and Saide 5, respectively. V, N, and C indicate variety, nitrogen application, and chemical regulation, respectively. Different letters in the same column indicate a significant difference between different treatments (*p* < 0.05). * and ** indicate significant differences at the 0.05 and 0.01 probability levels, respectively, and NS indicates no significant difference.

## Data Availability

The original contributions presented in the study are included in the article; further inquiries can be directed to the corresponding authors.

## Abbreviations

The following abbreviations are used in this manuscript:

PGR	Plant growth regulator
SPAD	Chlorophyll content
Pn	Net photosynthetic rate
Fv/Fm	Maximum quantum yield
RuBPCase	Ribulose-1,5-bisphosphate carboxylase/oxygenase
PEPCase	Phosphoenolpyruvate carboxylase
LAI	Leaf Area Index
PAL	Phenylalanine ammonia-lyase
TAL	Tyrosine ammonia-lyase
CAD	Cinnamyl alcohol dehydrogenase
4CL	4-coumarate:CoA ligase
GS	Glutamate synthetase
GDH	Glutamate dehydrogenase
GPT	Glutamate-pyruvate transaminase
DAA	Days after anthesis
